# Engineered Microneedle System Enables the Smart Regulation of Nanodynamic Sterilization and Tissue Regeneration for Wound Management

**DOI:** 10.1002/advs.202412226

**Published:** 2025-01-13

**Authors:** Shiyang Lin, Zhongqi Cui, Qingqiong Luo, Chen Li, Yue Zhang, Fengjiao Yang, Yichuan Chen, Chuansheng Xu, Yan Gao, Shasha Zhao, Fenyong Sun, Dandan Shen, Qi Wu, Shuo Shi

**Affiliations:** ^1^ Department of Laboratory Medicine School of Chemical Science and Engineering Shanghai Tenth People's Hospital of Tongji University Tongji University Shanghai 200092 P. R. China; ^2^ Department of Clinical Laboratory Medicine Shanghai Skin Disease Hospital, School of Medicine Tongji University Shanghai 200443 P. R. China; ^3^ Tongji University School of Medicine Tongji University Shanghai 200092 P. R. China; ^4^ Department of Clinical Laboratory Shanghai Children's Hospital Shanghai Jiao Tong University School of Medicine Shanghai 200062 P. R. China; ^5^ Key Laboratory of Endemic and Ethnic Diseases Ministry of Education Guizhou Medical University Guiyang 550004 P. R. China

**Keywords:** black phosphorus nanosheets, copper peroxide nanodots, engineered microneedle patch, nanodynamic sterilization, wound healing

## Abstract

The healing of bacterial biofilm‐infected wounds is a complex process, and the construction of emerging therapeutic modalities that regulate the microenvironment to magnify therapeutic effects and reduce biotoxicity is still highly challenging. Herein, an engineered microneedle (MN) patch is reported to mediate the efficient delivery of black phosphorus nanosheets (BP NSs) and copper peroxide nanodots (CP NDs) for dual nanodynamic sterilization and methicillin‐resistant staphylococcus aureus (MRSA)‐infected wound healing. Results demonstrate that the system can eliminate biofilm, reduce cytotoxicity, promote angiogenesis and tissue regeneration by the multiple advantages of chemodynamic therapy (CDT), enhanced photodynamic therapy (PDT), and improved degradation process from BP NSs to phosphate for promoting cell proliferation. Notably, the balance between excellent photodynamic stability and rapid degradability of BP NSs is maintained, and the improved degradation mechanism of BP NSs is vividly elucidated by density functional theory (DFT)‐based molecular dynamics (MD) calculations. Furthermore, the transcriptional changes of treated MRSA‐infected skin are studied using RNA‐seq technology to reveal the potential therapeutic mechanism. As envisaged, the proposed MN patch provides a safe, easy, also highly effective approach to achieve the temporal regulation of sterilization and tissue regeneration for bacterial biofilm‐infected wounds.

## Graphical abstract

An engineered microneedle patch can mediate the efficient delivery of black phosphorus nanosheets (BP NSs) and copper peroxide nanodots (CP NDs), maintain the balance between excellent photodynamic stability and rapid degradability of BP NSs, adapt the physiological healing process and achieve the temporal regulation of dual nanodynamic sterilization and tissue regeneration for bacterial biofilm‐infected wound management.

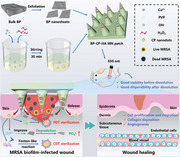

## 1 Introduction

To date, tissue infection with bacterial pathogens remains a major medical challenge, which may prolong the wound healing time and seriously affect patients' quality of life.^[^ ^1^, ^2^ ^]^ As one of the most common pathogens, methicillin‐resistant staphylococcus aureus (MRSA) can form dense biofilm through a special affinity between the carbohydrate‐binding proteins of bacteria and the galactose of wound, resulting in increased difficulty for wound healing.^[^ ^3^ ^]^ Besides the challenging issue of antibiotic resistance,^[^ ^4^, ^5^ ^]^ conventional and topical drug delivery methods face the problem of reduced bioavailability due to limited permeability.^[^ ^6^, ^7^, ^8^ ^]^ These unfavorable factors may complicate the therapeutic process of bacterial pathogens‐infected wounds. Thus, it is urgent to create more efficient, safer, easier therapies to address these difficulties.

As attractive devices containing miniaturized needles, microneedle (MN) patches can provide considerable benefits in various fields. During the past two or three decades, MN patches have been extensively explored as delivery or extraction tools for disease treatment,^[^ ^9^, ^10^, ^11^, ^12^, ^13^, ^14^ ^]^ physiological monitoring,^[^ ^15^, ^16^, ^17^, ^18^, ^19^, ^20^ ^]^ and so on. Very recently, there is a growing interest in antibacterial applications of MNs‐based implantable nanomedicine, as MNs can enhance drug penetration into bacterial biofilm and deep skin infection area. For example, a dissolvable MN patch containing Fe_2_C nanozyme and glucose oxidase can produce reactive oxygen species (ROS) for bacterial biofilm elimination and diabetic wound healing.^[^ ^21^ ^]^ The ultrasound‐triggered MN patch can mediate the transdermal delivery of ZnTCPP@ZnO sonosensitizer and overload of ROS for bacterial infection acne repair.^[^ ^22^ ^]^ Despite some achievements, many current investigations mainly focus on creating highly toxic ROS to eradicate bacterial pathogens, while ignoring dose‐dependent toxicity to healthy tissues and organs. What's more, it remains a tremendous challenge to adapt the physiological healing process and realize the temporal control or regulation of sterilization and tissue regeneration for ideal wound management. Therefore, more elaborately designed/engineered MN patches with reduced biotoxicity and adaptive temporal modulation are highly expected to make a huge difference in medical applications including the antibacterial region.

Black phosphorus nanosheets (BP NSs), as a member of the multifunctional 2D materials family, have been incorporated into several nanosystems for biomedical practices.^[^ ^23^, ^24^ ^]^ Typically, BP NSs can act as desired carriers or photosensitizers for drug delivery, photothermal and photodynamic therapies due to its fascinating puckered structure and semiconductor property.^[^ ^25^, ^26^, ^27^, ^28^ ^]^ However, it has been reported that BP NSs feature inherent size‐relied cytotoxicity^[^ ^29^, ^30^ ^]^ and slow degradation performance especially in acidic environments,^[^ ^31^ ^]^ creating potential biosafety concerns in some therapeutic applications with acidic and hypoxia microenvironment, such as tumor therapy^[^ ^32^, ^33^ ^]^ and some skin infections.^[^ ^34^, ^35^ ^]^ Therefore, it is crucial to appropriately accelerate the degradation process of BP NSs in some cases. Nevertheless, rapid degradation can compromise the stability of BP NSs that plays a vital role in ensuring remarkable therapeutic effects, and it is extremely difficult to solve the conflict between great stability and rapid degradation of BP NSs. Inspired by the fact that Cu^2+^ can accelerate the degradation of BP NSs,^[^ ^27^ ^]^ it is estimated that this conflict can be broken by adding some copper peroxide nanodots (CP NDs), and then further encapsulated by a dry dissolvable MN patch. Specifically, the MN patch can protect BP NSs from degradation to ensure the long‐term stability before dissolving, and the degradation process of BP NSs can be improved when meeting with Cu^2+^ released from decomposed CP NDs in liquid state after the MN patch is dissolved. What's more, the marriage of BP NSs and CP NDs can achieve dual nanodynamic therapy including chemodynamic therapy (CDT) and photodynamic therapy (PDT). Therefore, this simple engineered MN system may meet the strict requirements on biodegradation and biocompatibility in the field of biomedicine, and further promote the application process of nanomaterials.

**Scheme 1:**
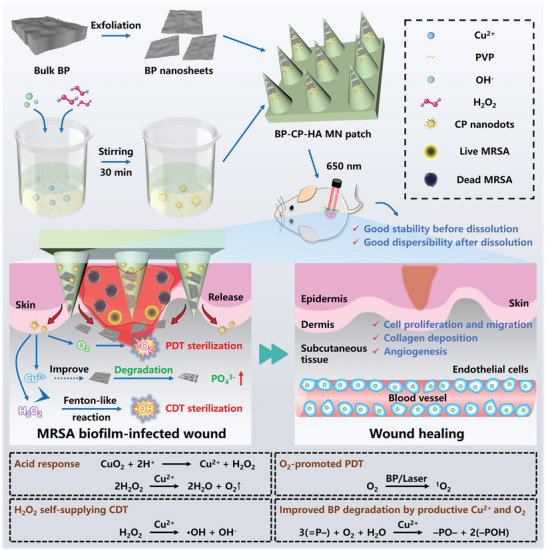
Schematic diagram of the preparation of BP‐CP‐HA MN patch and the mechanism of dual nanodynamic sterilization and tissue regeneration for MRSA biofilm‐infected wound healing.

In this work, a MN patch system (abbreviated as BP‐CP‐HA MN patch) was elaborately designed by the integration of two nanomaterials (BP NSs, CP NDs) and dissolvable sodium hyaluronate (HA) for implantable nanomedicine of MRSA biofilm‐infected wound healing under a 650 nm laser irradiation (**Scheme** **1**). Once the BP‐CP‐HA MNs penetrated the wound area, BP NSs and CP NDs would be released with the gradual dissolution of HA matrix. On one hand, CP NDs could produce H_2_O_2_ and O_2_ in the acidic microenvironment, thereby increasing the substrate concentrations of CDT and PDT. On the other hand, the degradation process from BP NSs to phosphate could be accelerated by Cu^2+^ to reduce the biotoxicity of BP NSs and promote cell proliferation. As a result, both in vitro and in vivo experiments demonstrated the amplified antibacterial activity and improved biocompatibility of such a composite MN system for better biofilm elimination, angiogenesis, and wound healing. Therefore, this multifunctional BP‐CP‐HA MN patch may meet the strict needs in biomedical engineering of biodegradation and biocompatibility, and act as a new paradigm for biomedical application.

## 2 Results and Discussion

### 2.1 Synthesis and Characterization of BP NSs and CP NDs

**Figure 1:**
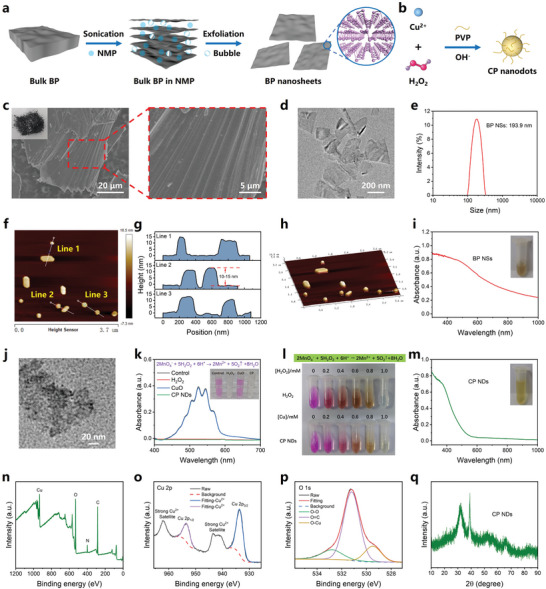
Synthesis and characterization of BP NSs and CP NDs. Diagrammatic sketch of the synthesis of a) BP NSs and b) CP NDs. c) SEM image of bulk BP. The local magnified SEM image showing the multilayer structure of bulk BP. Inset: representative photograph of bulk BP. d) TEM image, e) dynamic light scattering (DLS) analysis, f) 2D AFM image, g) corresponding AFM‐measured thickness, h) 3D AFM image, and i) UV‐Vis‐NIR spectra of BP NSs. j) TEM image of CP NDs. k) KMnO_4_‐based fading reaction demonstrating the generation of H_2_O_2_ from CP NDs in an acidic environment, thus proving the presence of peroxo groups in CP NDs. l) Concentration‐dependent KMnO_4_ fading reaction by CP NDs and H_2_O_2_. m) UV‐Vis‐NIR spectra of CP NDs. XPS spectra for n) CP NDs, o) Cu 2p, and p) O 1s. q) XRD pattern of CP NDs.

BP NSs were synthesized from bulk BP by the sonication‐assisted N‐methyl‐2‐pyrrolidone (NMP) exfoliation strategy (**Figure** **1**a). Scanning electron microscope (SEM) analysis demonstrated the commercial bulk BP featured typical multilayer stacked morphology (Figure 1c). After exfoliation, transmission electron microscope (TEM) image showed the obvious 2D sheet‐like nanostructure (Figure 1d), and the hydrodynamic size was around 193.9 nm (Figure 1e). The obtained BP NSs from bulk BP could be indexed into the orthorhombic BP crystalline structure with the standard JCPDS card No. 73–1358 (Figure S1a, Supporting Information). Three observed Raman peaks in Raman spectra (Figure S1b, Supporting Information) were assigned to the out‐of‐plane vibrational A_g_^1^ mode, in‐plane B_2g_ and A_g_^2^ modes, and the slightly shifted peak positions of BP NSs might be related with the variation of thickness.^[^ ^36^, ^37^, ^38^ ^]^ Furthermore, the atomic force microscopy (AFM) technique revealed BP NSs were of desirable dispersion (Figure 1f, h), and the thickness was ≈10–15 nm (Figure 1g). Valuably, the obtained BP NSs displayed broad optical absorptions, showing a great potential to act as desired photosensitizers (Figure 1i).

CP NDs were synthesized by a simple PVP‐assisted dispersion method (Figure 1b). TEM image showed that the tiny black dots (CP NDs) were surrounded by irregular nebulosity (PVP coating) (Figure 1j). KMnO_4_‐based fading reaction (the reduction of MnO_4_^−^ to colorless Mn^2+^ by H_2_O_2_) demonstrated the generation of H_2_O_2_ from CP NDs in an acidic environment, thus proving the presence of peroxo groups in CP NDs (Figure 1k,l). Unlike BP NSs, CP NDs showed weak absorptions from 600 to 1000 nm (Figure 1m). The emergence of N 1s peak in XPS spectra confirmed that CP NDs were coated by PVP (Figure 1n). The XPS spectra of Cu 2p and O 1s proved the presence of Cu^2+^ and peroxo groups in CP NDs (Figure 1o,p). Notably, the XPS spectra of commercial CuO nanoparticles were analyzed to further prove the successful synthesis of PVP‐coated CP NDs (Figure S2a‐c, Supporting Information). Besides, the X‐ray powder diffraction (XRD) diffraction peaks of CP NDs were weakened because of the amorphous PVP coating (Figure 1q).

### 2.2 Chemodynamic Performance, O_2_ Production, Promoted Photodynamic Performance, and Improved Degradation of BP NSs and CP NDs

**Figure 2:**
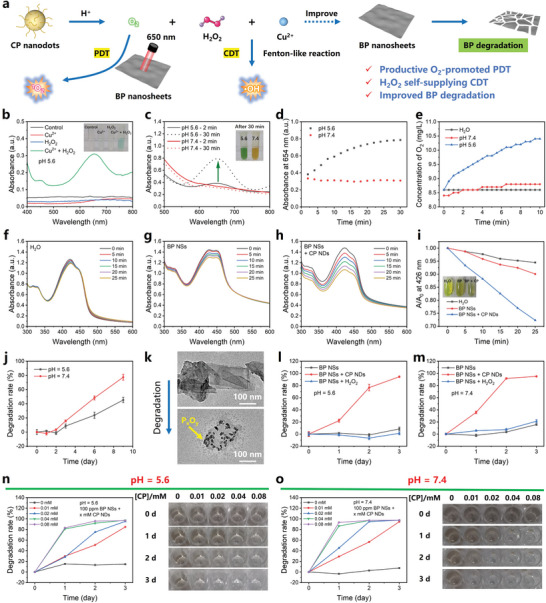
Various performances of BP NSs and CP NDs. a) Schematic illustration showing acid‐responsive CDT and O_2_ production of CP NDs, O_2_‐promoted BP PDT and improved BP degradation by Cu^2+^. b) Absorption spectra and photographs (inset) of TMB solution treated with H_2_O, Cu^2+^, H_2_O_2_, Cu^2+^ + H_2_O_2_ (pH = 5.6). c) Absorption spectra, photographs (inset), and d) time‐dependent absorption changes (654 nm) of TMB solution treated with CP NDs at pH 5.6 and pH 7.4. e) O_2_ concentration of CP NDs aqueous solution at pH 5.6 and pH 7.4. Time‐dependent absorption spectra of DPBF treated with f) H_2_O, g) BP NSs, h) BP NSs + CP NDs under laser irradiation (650 nm, 0.668 W cm^−2^). i) Normalized absorbance of DPBF at 426 nm from figure f‐h. j) Degradation of BP NSs under pH 5.6 and pH 7.4 conditions (*n* = 3). k) TEM images showing the degradation of BP NSs. Degradation of BP NSs treated with H_2_O, H_2_O_2_, CP NDs under l) pH 5.6 and m) pH 7.4 conditions (*n* = 3). Degradation of BP NSs incubating with different concentrations of CP NDs and the corresponding photographs within 3 days under n) pH 5.6 and o) pH 7.4 conditions.

It is critical to reveal the exact performances of BP NSs and CP NDs for better theoretical understanding and practical application. As shown in **Figure** **2**a, CP NDs were expected to be decomposed into Cu^2+^, H_2_O_2_, and O_2_. On one hand, CP NDs could produce ⋯OH by H_2_O_2_ self‐supplying CDT process. On the other hand, the products from decomposed CP NDs could promote the highly O_2_‐dependent PDT process of BP NSs to generate ^1^O_2_ for short‐term therapeutic effect, and gradually oxidize and eliminate BP NSs for long‐term therapeutic safety. Considering the inherent acidic microenvironment (pH values of 4.5‐6.5) within the biofilm due to the metabolic activity of the bacteria and host immune response,^[^ ^39^, ^40^, ^41^ ^]^ a pH value of 5.6 (between 4.5 and 6.5) was chosen here to simulate the wound microenvironment. Colorless 3,3′,5,5′‐tetramethylbenzidine (TMB) was used as the probe of •OH, and only Cu^2+^ + H_2_O_2_ group presented an apparent blue color (oxidized TMB) triggered by the Fenton‐like reaction (Figure 2b). Therefore, the acid‐responsive H_2_O_2_ generation and continuous •OH generation were firmly proved by the colorimetric analysis and absorption spectra at 654 nm (Figure 2c,d). O_2_ production ability of CP NDs was assessed by the dissolving oxygen levels, which was also caused by acid‐related decomposition from CP NDs (Figure 2e). Next, yellow 1,3‐diphenylisobenzofuran (DPBF) was used to monitor the ^1^O_2_ generation by a Diels‐Alder 1,4‐cycloaddition reaction (yellow fading). The results showed that the absorption intensities ≈426 nm of BP NSs group and BP NSs + CP NDs group were gradually decreased with the increase of laser irradiation time, demonstrating the generation of ^1^O_2_ (Figure 2f‐i). Valuably, the fastest absorption decay in BP NSs + CP NDs group revealed the most efficient ^1^O_2_ generation, which was promoted by CP NDs. In fact, BP NSs as desired photosensitizers have inherent cytotoxicity and usually degrade slowly. Inspired by the fact that Cu^2+^ can accelerate the degradation of BP NSs,^[^ ^27^ ^]^ it is estimated that the degradation problem of BP NSs can be solved by adding appropriate amount of CP NDs. As shown in Figure 2j, the results proved the slow degradation process of BP NSs, and faster degradation rate in neutral solution than acidic solution was attributed to OH^−^‐initiated structural destruction.^[^ ^31^ ^]^ TEM revealed the degradation process of BP NSs. It was noted that some small nanoparticles were found to be attached on the surface of BP NSs, which was due to the surface oxidation of BP NSs (Figure 2k). After mixed with CP NDs, the degradation speed of BP NSs was accelerated (Figure 2l,m), which could be easily controlled by adjusting the relative concentrations between BP NSs and CP NDs (Figure 2n,o). The possible factors to accelerate the degradation of BP NSs were further explored through incubating BP NSs with different components (H_2_O, CP NDs, Cu^2+^, H_2_O_2_, Cu^2+^ + H_2_O_2_). The results indicated that the degradation of BP NSs was mainly influenced by Cu^2+^ (Figure S3a‐c, Supporting Information).

### 2.3 Fabrication, Characterization, and Degradation Behavior of BP‐CP‐HA MN Patch

**Figure 3:**
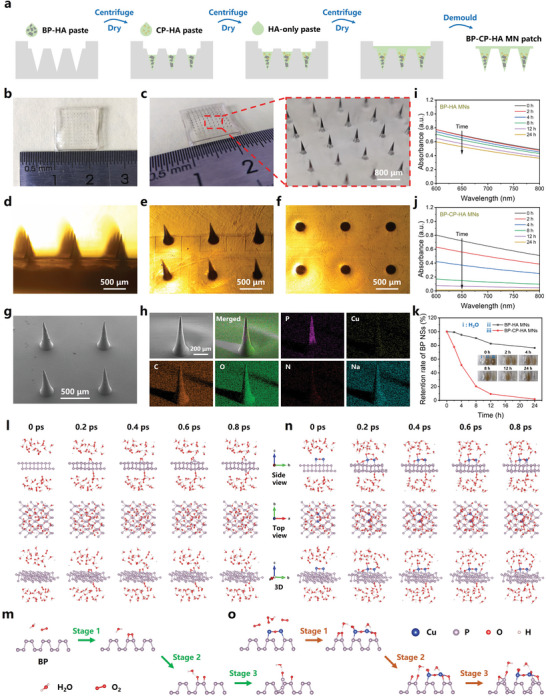
Fabrication, characterization, and degradation behavior of BP‐CP‐HA MN patch. a) Schematic diagram of the fabrication process of BP‐CP‐HA MN patch. Representative b) photograph and c) macro photograph of BP‐CP‐HA MN patch. The local magnified image showing the needle arrays. d‐f) Microscopic images of BP‐CP‐HA MNs from different view directions. g) SEM image of BP‐CP‐HA MNs. h) Elemental mapping of a single BP‐CP‐HA MN. Absorption spectra of i) BP‐HA MNs and j) BP‐CP‐HA MNs after dissolving in deionized water for different time points. k) Retention rates of BP NSs calculated by the decreased absorption at 650 nm in two groups. Inset: photographs of BP‐HA MNs solution and BP‐CP‐HA MNs solution, deionized water was used as the control group. l) Representative reaction‐degradation path and m) corresponding possible degradation mechanism of monolayer BP in ambient system (310 K) contains H_2_O and O_2_. Stage 1: P–O bond‐formation (physisorption of O_2_ on the surface of BP NSs); Stage 2: O–O bond‐breaking (from physisorption to chemisorption of O_2_ on the surface of BP NSs); Stage 3: P–P bond‐breaking. n) Representative reaction‐degradation path and o) corresponding possible degradation mechanism of monolayer BP in ambient system (310 K) contains H_2_O, O_2_, and Cu^2+^. Stage 1: increased physisorption of O_2_; Stage 2: from physisorption to chemisorption of O_2_ on the surface of BP NSs; Stage 3: unstable P–P bond as a prerequisite for the structural collapse of BP NSs.

Sodium hyaluronate (HA) was employed as the matrix material to form the MN patches. The dispersion and degradation of HA‐involved system were first characterized. The results showed that HA could improve the dispersibility of nanomaterials and slightly inhibit the degradation of BP NSs (Figures S4 and S5, Supporting Information), which meant the HA‐involved system might feature better biocompatibility, and higher concentrations of CP NDs could be employed to enhance the therapeutic effects while maintaining the stability of BP NSs. Here, four types of MN patches (HA MNs, BP‐HA MNs, CP‐HA MNs, BP‐CP‐HA MNs) were fabricated for comparative study. To optimize the relative contents of BP NSs and CP NDs, different concentrations of CP‐HA paste were used to fabricate CP‐HA MNs and BP‐CP‐HA MNs (Figures S6 and S7, Supporting Information), all MN patches showed arranged needle structure. After dissolving these MN tips, the released BP NSs showed gradient degradation based on different concentrations of CP NDs, indicating the controllable degradation of BP NSs could be easily achieved by adjusting the concentrations of CP‐HA paste in the process of fabricating the MN patches. Notably, BP‐CP‐HA MNs fabricated with 36 mM CP NDs showed suitable concentration (Figure S6, Supporting Information) and degradation speed within 24 h (Figure S8, Supporting Information), and were adopted for subsequent research. **Figure** **3**a shows the fabrication process of BP‐CP‐HA MN patch containing BP NSs and CP NDs. The 10 × 10 needle arrays were located in the back layer of 12.5 mm × 12.5 mm (Figure 3b,c). The conical needles were ≈600 µm in height, 230 µm in base diameter, and 1 mm in distance between two nearest needles (Figure 3d–f). SEM image further demonstrated the conical structure, and elemental mapping analysis proved the successful loading of nanomaterials according to the presence of P and Cu elements (Figure 3g,h). According to Vis‐NIR spectra and inductively coupled plasma optical emission spectrometry (ICP‐OES) analysis, the contents of BP NSs and CP NDs in per MN patch were calculated to be ≈23.6 µg and 22.3 nmol, respectively (Figure S9, Supporting Information). Besides, other three types of MN patches were also fabricated, showing good needle structures (Figures S10‐S12, Supporting Information).

Due to the accelerated degradation of BP NSs induced by CP NDs, the long‐term storage of nanomaterials after being encapsulated by MNs is an important consideration for practical applications. Encouragingly, BP‐CP‐HA MNs still kept black after a long time of observation, indicating that the dry MN carrier could increase the stability of nanomaterials (Figure S13, Supporting Information). Interestingly, BP NSs could be gradually degraded and the degradation process could be accelerated by CP NDs after dissolving MNs, which were further quantified according to the decreased Vis‐NIR absorbance intensity (Figure 3i‐k). To confirm the accelerated degradation behavior of BP NSs interacting with CP NDs, density functional theory (DFT)‐based molecular dynamic (MD) simulations were performed to reveal the reaction mechanism of BP NSs + CP NDs + H_2_O + O_2_ system (monolayer BP, 310 K). However, the results showed that CP NDs failed to combine well with BP NSs, indicating that complete (undecomposed) CP NDs might not promote the degradation process of BP NSs (Figure S14, Supporting Information). Given the above phenomenon that the degradation process of BP NSs could be accelerated by Cu^2+^ (Figure S3, Supporting Information), the reaction mechanism of Cu^2+^ (from CP NDs) on promoting BP degradation was further evaluated (Figure S15, Supporting Information). The concrete MD snapshots for BP NSs + H_2_O + O_2_ (Figure S16, Supporting Information), and BP NSs + Cu^2+^ + H_2_O + O_2_ (Figure S17, Supporting Information) are presented. Unlike complete CP NDs, Cu^2+^ could combine well with BP NSs, which provided the precondition for accelerated BP degradation. It is worth noting that a three‐step reaction mechanism in BP NSs + H_2_O + O_2_ group is revealed: (i) P–O bond‐formation caused by the thermodynamically driven combination of three‐coordinated ═P─ with O_2_. (ii) O–O bond‐breaking caused by the transformation of a low‐barrier hydrogen from H_2_O to ═P─O─O─P═. (iii) P─P bond‐breaking to form P─O and P─OH. (Figure 3l,m). In the presence of Cu^2+^, on one hand, Cu^2+^ can increase O_2_ enrichment on BP NSs to promote the degradation process (Figure 3n,o). On the other hand, as Cu^2+^ moves in BP structure, the structure integrity of BP NSs can be further disrupted (Figure S17, Supporting Information). Consequently, the whole degradation process of BP NSs can be described as the reaction mechanism of 3(═P─) + O_2_ + H_2_O → –PO– + –POH + = P– + OH^−^ → –PO– + 2(–POH). Notably, even though complete CP NDs may be friendly to maintain the structural integrity, increased O_2_ from decomposed CP NDs can directly promote the degradation of BP NSs.

To further prove that there were adequate BP NSs and CP NDs available in the system for efficient PDT and CDT, the H_2_O_2_ self‐supplying chemodynamic ability of the MN system was also validated by the typical colorimetric method based on TMB. As a result, a pale blue solution with the absorption peak at 654 nm was observed after the reaction between CP‐HA MN leaching solution and TMB‐contained PBS solution, proving the copper ions in the system were sufficient to produce a certain level of •OH (Figure S18, Supporting Information). DPBF was resorted to quantitatively analyze the radical,^[^ ^42^ ^]^ and DPBF‐based ^1^O_2_ detection was evaluated according to the relative concentration of ≈0.94 mmol CP NDs g^−1^ BP NSs. The results showed that compared with bare BP NSs (with a reference quantum yield of ≈0.91),^[^ ^26^ ^]^ BP NSs supplemented with CP NDs exhibited enhanced photodynamic efficiency with an increased ^1^O_2_ quantum yield of ≈14%, which emphasized this novel strategy for broadening the application of BP NSs in the field of biomedicine (Figure S19, Supporting Information). What's more, electron spin resonance (ESR) was chosen to further confirm the types of ROS by using 2,2,6,6‐Tetramethylpiperidine (TEMP) as ^1^O_2_ trapping agent and 5,5‐dimethyl‐1‐pyrroline N‐oxide (DMPO) as •OH trapping agent. Compared with the BP‐HA MNs group, the intensity of the specific peak (a triplet peak 1:1:1) in the BP‐CP‐HA MNs group was significantly increased, indicating the photodynamic efficiency of BP NSs for ^1^O_2_ generation could be enhanced by CP NDs (Figure S20, Supporting Information). Besides, the generation of •OH (a quadruple peak 1:2:2:1) induced by Fenton‐like reaction in the CP‐HA MNs group indicated the good chemodynamic performance of CP NDs (Figure S21, Supporting Information). What's more, GSH depletion of MNs was further investigated to support ROS‐based therapeutic applications,^[^ ^43^, ^44^, ^45^, ^46^ ^]^ and only CP NDs‐involved MNs groups showed weakened absorption peak of DTNB, indicating the GSH depletion by CP NDs (Figure S22, Supporting Information). Furthermore, considering the long‐term GSH depletion, concentration and time‐dependent GSH depletion by CP NDs were also proven to support antimicrobial practice (Figure S23, Supporting Information).

Porcine skin was chosen to test the skin‐insertion capacity of BP‐CP‐HA MNs to ensure the nanomedicine delivery ability for practical applications. After pressing a BP‐CP‐HA MN patch and removing its back layer, microchannels filled with released materials could be observed (Figure S24, Supporting Information), demonstrating MNs featured sufficient strength to penetrate biological tissues. Furthermore, the dissolution ability was tested using both porcine skin and PBS solution, indicating MNs were capable of releasing nanomedicines within two minutes (Figures S25a and S26a, Supporting Information). In addition, the HA back layer could provide strong adhesion after contacting with liquid (Figures S25b and S26b, Supporting Information), which might protect the wound area against reinfection during the treatment. To comprehensively exclude the photothermal effect, the corresponding photothermal experiments on MN leaching solution, MN patches, and porcine skin‐inserted MN patches were conducted (Figure S27, Supporting Information). Under the laser irradiation (650 nm, 0.668 W cm^−2^, 10 min), only BP‐HA MNs groups and BP‐CP‐HA MNs groups showed slight photothermal performances due to the presence of BP NSs. As an example, the temperature of BP‐CP‐HA MNs rose slightly from 27.5 to 30.6 °C even though in the dry state, which was far from the hyperthermia for bacteria killing. Therefore, the photodynamic performance of BP‐CP‐HA MNs is dominant rather than the photothermal performance under the conditions of this study.

### 2.4 In Vitro Cytotoxicity, Cell Proliferation, Migration, and Vascularization Evaluation

**Figure 4:**
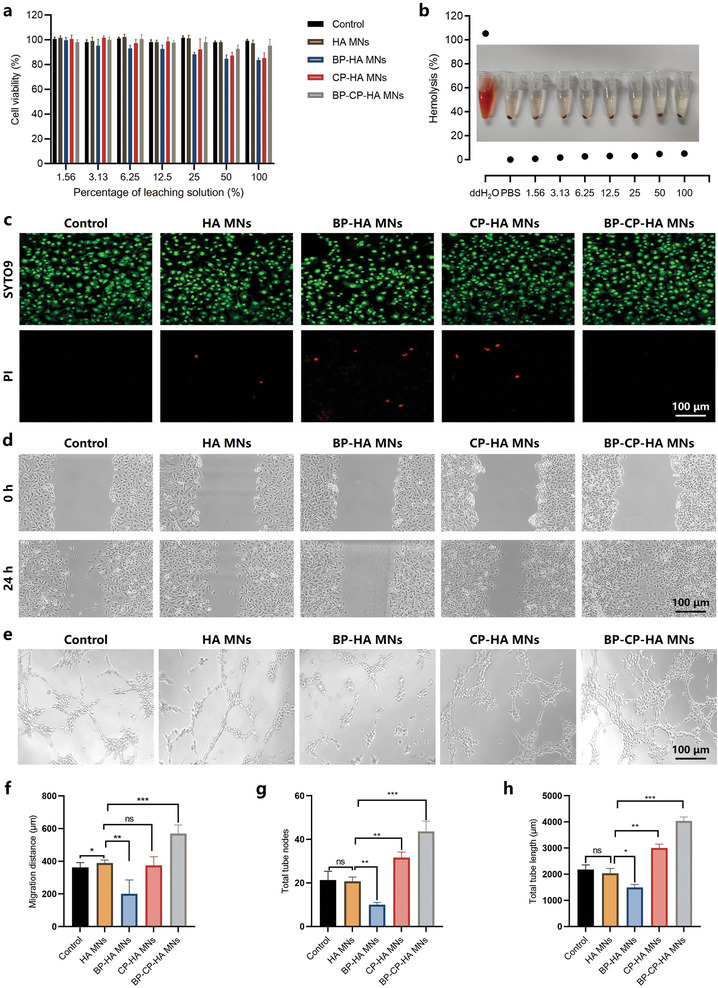
In vitro cytotoxicity, cell proliferation, migration, and vascularization evaluation. a) Viabilities of HUVECs incubated with different MN leaching solutions at different dilution ratios for 24 h. b) Hemolysis rate of red blood cells after co‐incubation with different MN leaching solutions (50%). c) Live/dead cell assay, d) scratch widths, e) tube formation ability, f) migration distance, g) number of tube nodes, and h) tube length of HUVECs after co‐incubation with different MN leaching solutions (50%). Data are presented as mean ± SD (*n* = 3), *p < 0.05, **p < 0.01, and ***p < 0.001, ns: no significance.

Considering the practical application of BP‐CP‐HA MN patch, the biosafety deserved to be evaluated using MN leaching solution. Human umbilical vein endothelial cells (HUVECs) were incubated with different MN leaching solutions at different dilution ratios for 24 h. The CCK‐8 assay showed that the BP‐HA MNs group and CP‐HA MNs group caused lower cell viabilities, while the corresponding BP‐CP‐HA MNs exhibited negligible cytotoxicity, which might be attributed to the accelerated degradation from toxic BP NSs to nontoxic phosphate by Cu^2+^ (**Figure** **4**a). Meanwhile, the remarkable green fluorescence in live/dead cell assay confirmed the great biocompatibility of BP‐CP‐HA MNs (Figure 4c). Furthermore, the co‐incubation of erythrocytes and MN leaching solutions did not cause significant hemolysis (Figure 4b). In scratch assay, the BP‐CP‐HA MNs group showed the minimum scratch width in all groups, while the BP‐HA MNs group showed the maximum distance, indicating the accelerated degradation of BP NSs by Cu^2+^ could effectively promote cell proliferation and migration (Figure 4d,f). Besides, the cell proliferation and migration were evaluated by adding extra phosphoric acid. With the increase of P element, cell proliferation and migration were accelerated (Figure S28, Supporting Information), which further supported the hypothesis that phosphate could promote cell proliferation. What's more, the cell proliferation effect of the system after laser irradiation was also evaluated (Figure S29, Supporting Information). The results showed that even though the enhanced dual nanodynamic effects could slightly inhibit cell proliferation in a short period of time, the long‐term cell proliferation could be expected with the degradation from toxic BP NSs to nontoxic phosphate, and the massive death of bacteria could also contribute to the further healing of the wound. After co‐incubation for 24 h, tubular structures were observed due to the interconnections of HUVECs (Figure 4e). The number of tube nodes and tube length were analyzed to reveal the ability of HUVECs to form tubes. After different treatments, the vascularization in the HA MNs group was similar to that in the control group, demonstrating HA matrix alone had no effect on forming tubes. It was noted that the vascularization in CP‐HA MNs group was enhanced compared to the control group, which might be assigned to the biological role of copper ions. Obviously, the BP‐HA MNs group failed to form tubes, while the BP‐CP‐HA MNs group showed maximum tube nodes and longest tube length than any other groups, firmly demonstrating the great tube forming effect of such a composite system (Figure 4g,h). Overall, these findings underscore BP‐CP‐HA MNs feature great biocompatibility, and a good capability to promote cell proliferation, migration, and vascularization in vitro.

### 2.5 In Vitro Antibacterial Assay

**Figure 5:**
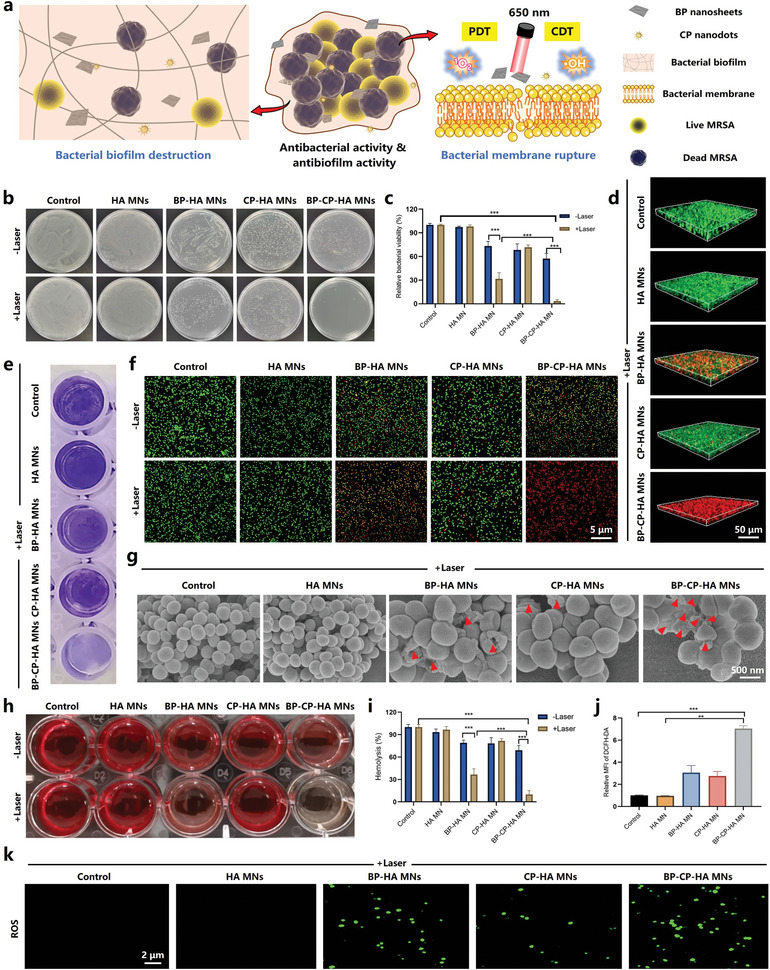
In vitro antibacterial assay. a) Schematic illustration showing the antibacterial and antibiofilm activity of BP NSs/CP NDs nanosystem. b) Representative images of MRSA colonies in the agar plates after different treatments and c) the corresponding bacterial viabilities. d) 3D reconstructions of the bacterial live (green fluorescence)/dead (red fluorescence) staining of MRSA biofilm. e) Macroscopic MRSA biofilm images with crystal violet staining. f) Live (green fluorescence)/dead (red fluorescence) staining and g) SEM images of MRSA with different treatments. h, i) Hemolysis of red blood cells after co‐incubation with MRSA in different groups. j) ROS levels and k) fluorescence images of DCFH‐DA‐stained MRSA after different treatments. Data are presented as mean ± SD (*n* = 3), *p < 0.05, **p < 0.01, and ***p < 0.001.

Besides the accelerated biodegradation performance, this research also highlighted the remarkable ROS amplification function through the combined action of PDT and CDT in short term to prompt the comprehensive exploration of BP‐CP‐HA MNs in antibacterial potential. Considering the significant toll of MRSA in the world,^[^ ^47^ ^]^ controlling and reducing the burden of this bacterial pathogen is critical. Therefore, MRSA was chosen to assess the antibacterial and antibiofilm activity here (**Figure** **5**a). The standard colony counting results (Figure 5b,c) and live/dead fluorescence images (Figure 5f) illustrated the HA matrix almost had no antibacterial activity. MRSA exhibited relatively moderate bacterial death in the BP‐HA MNs, CP‐HA MNs and BP‐CP‐HA MNs groups. After laser irradiation, the BP‐HA MNs + Laser and BP‐CP‐HA MNs + Laser groups showed significantly increased bacterial death, indicating the remarkable PDT sterilization of BP NSs. Importantly, the antibacterial performance in CDT/PDT combination (BP‐CP‐HA MNs + Laser group) surpassed that of individual CDT (CP‐HA MNs group) or PDT (BP‐HA MNs + Laser group), supporting the hypothesis that the enhanced antibacterial activity could be achieved by this ROS‐amplified MN system. 3D live/dead fluorescence (Figure 5d) and crystal violet staining (Figure 5e) were further used to confirm antibiofilm activity. The results showed that the biofilm in Control, HA MNs, CP‐HA MNs groups remained whole or mostly viable and structurally intact even though with the laser irradiation. In contrast, the BP‐HA MNs + Laser and BP‐CP‐HA MNs + Laser groups disrupted the tight biofilm with more dead bacteria, and BP‐CP‐HA MNs could achieve the best antibiofilm activity with the laser irradiation. Hemolysin is one of the main pathogenic factors, and inhibiting the secretion of hemolysin is also a representative indicator to reflect the antibacterial activity of antimicrobial drug.^[^ ^48^ ^]^ The treated bacteria were centrifuged, and the ability of bacterial hemolysin in the supernatant medium to lyse red blood cells was observed. Encouragingly, both qualitative (Figure 5h) and quantitative (Figure 5i) evaluations demonstrated the hemolysin secreted by MRSA in BP‐CP‐HA MNs + Laser group was significantly inhibited, further highlighting the antibacterial potential of BP‐CP‐HA MNs. SEM imaging was employed to visualize the morphological changes of MRSA. As shown in Figure 5g, MRSA displayed a relatively smooth surface with intact membrane structure in Control and HA MNs with laser, indicating laser irradiation alone or the matrix material of MNs failed to cause antibacterial effect. By contrast, shrunken membrane structures were found after treated with leaching solutions of BP‐HA MNs, CP‐HA MNs, BP‐CP‐HA MNs under the laser irradiation, verifying the damage or death of MRSA. What's more, BP‐CP‐HA MNs induced the most dead/abnormal MRSA and highest ROS level (Figure 5j,k), further validating the enhanced effect of PDT and CDT. Put it another way, malondialdehyde (MDA) as a vital metabolite of lipid peroxidation^[^ ^49^ ^]^ was also quantified to reflect the oxidative stress damage. Encouragingly, the MDA accumulation results were consistent with the ROS measurement results, and BP‐CP‐HA MNs greatly facilitated the MDA accumulation under the laser irradiation (Figure S30a, Supporting Information). It was reported that ROS‐mediated MDA accumulation could damage the bacterial respiratory chain and inhibit ATP synthesis,^[^ ^49^, ^50^ ^]^ so the activities of bacterial respiratory chain complexes and ATP levels were further examined, and the results showed that the ATP level and the activities of complexes I, II, and III were remarkably decreased in BP‐CP‐HA MNs‐treated MRSA isolates under the laser irradiation (Figure S30b, c, Supporting Information). Furthermore, considering the GSH‐rich environment, GSH content evaluation in bacteria was also detected to support ROS‐based antimicrobial applications.^[^ ^51^, ^52^ ^]^ The results showed that BP‐HA MNs, CP‐HA MNs, BP‐CP‐HA MNs could reduce the GSH content, while BP‐CP‐HA MNs group induced the most loss of GSH (Figure S31, Supporting Information), proving that GSH depletion could be efficiently achieved by the MN system under laser irradiation. Thus, BP‐CP‐HA MNs could cause ROS production (along with GSH depletion), hemolysin inhibition, membrane damage, decreased ATP levels and activities of respiratory chain complexes (especially complexes I, II, and III), ultimately leading to the death of MRSA. All in all, these results highlight BP‐CP‐HA MNs involve self‐reactive and laser‐excited ROS amplification modes for efficient sterilization and biofilm elimination.

### 2.6 In Vivo Therapeutic Assessment

**Figure 6:**
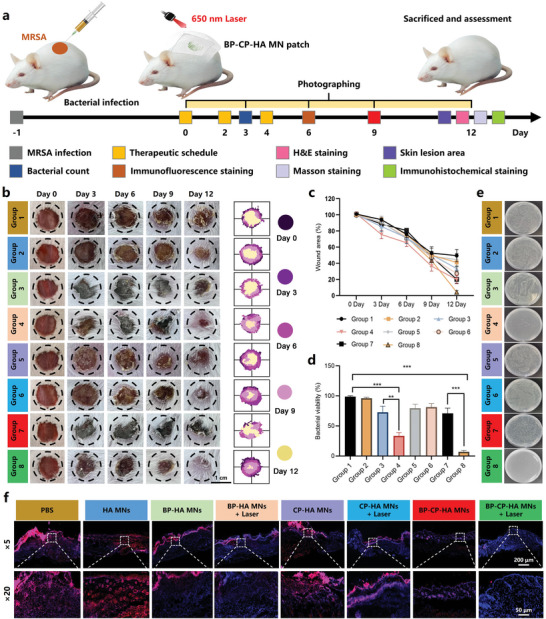
In vivo therapeutic assessment. a) Schematic design of MRSA‐infected wound establishment and experimental procedures. b) Photographs and simulated changes of the wound areas with different periods in different groups. Group 1: PBS; Group 2: HA MNs; Group 3: BP‐HA MNs; Group 4: BP‐HA MNs + Laser; Group 5: CP‐HA MNs; Group 6: CP‐HA MNs + Laser; Group 7: BP‐CP‐HA MNs; Group 8: BP‐CP‐HA MNs + Laser. c) Change rate of wound area after different treatments (*n* = 5). d) Statistics and e) representative photographs of MRSA from the wound areas in different groups. f) Frozen section staining showing the ROS level of wound tissues after different treatments. Data are presented as mean ± SD (*n* = 5), *p < 0.05, **p < 0.01, and ***p < 0.001.

Encouraged by the persuasive antibacterial and antibiofilm activity in vitro, we further conducted comprehensive experiments and analyses to assess the therapeutic efficacies of BP‐CP‐HA MNs in vivo by creating a MRSA biofilm‐infected skin wound model in mice (**Figure** **6**a). Eight groups (PBS, HA MNs, BP‐HA MNs, BP‐HA MNs + Laser, CP‐HA MNs, CP‐HA MNs + Laser, BP‐CP‐HA MNs, BP‐CP‐HA MNs + Laser) were chosen and the progress of wound healing was monitored on day 3, day 6, day 9 and day 12. Representative photographs (Figure 6b), statistics of wound areas (Figure 6c) and bacterial viability (Figure 6d,e) revealed that wound healing and antibacterial effect (96.43 ± 7.64%) in BP‐CP‐HA MNs + Laser group (a decrease around 97.90 ± 4.44% in wound area on day 12) far surpassed those in any other groups. On day 6, skin from the wound sites was collected for histological analyses to evaluate the amount of ROS content, in which dihydroethidium (blue fluorescence) could be oxidized by ROS to form ethidium oxide (red fluorescence).^[^ ^53^ ^]^ The results showed that the red fluorescence of the BP‐CP‐HA MNs + Laser group was obviously lower than that of any other groups (Figure 6f; Figure S32, Supporting Information), indicating that the ROS level of the wound site was reduced during the wound healing stage after the sterilization process. Furthermore, the blood routine/biochemical indicators and the main organs (heart, liver, spleen, lung and kidney) showed no significant blood toxicity and pathological changes (Figures S33‐S35, Supporting Information). Thus, the engineered BP‐CP‐HA MNs featured short‐term antibacterial activity under the laser irradiation, and long‐term wound healing ability without causing significant side effects.

**Figure 7:**
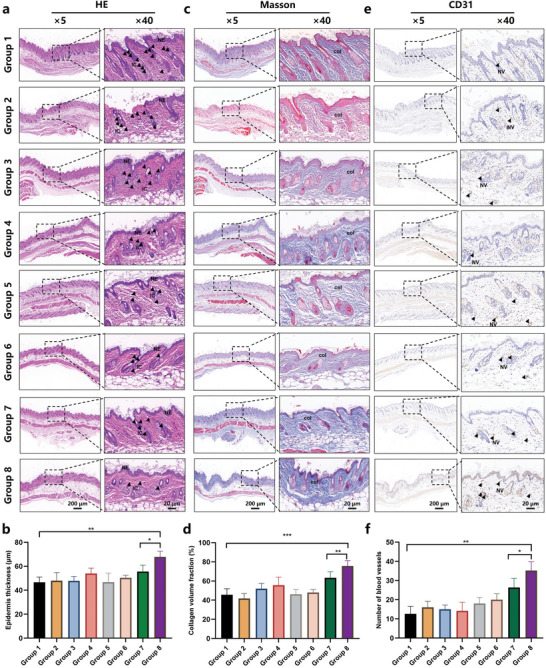
Investigation on wound healing process. a) H&E staining images, and b) thickness of the newly formed epidermis showing the wound healing effects. c) Masson's staining images, and d) collagen volume fraction showing the collagen deposition effects. e) CD31 immunohistochemistry staining images, and f) number of blood vessels showing the angiogenesis effects. Group 1: PBS; Group 2: HA MNs; Group 3: BP‐HA MNs; Group 4: BP‐HA MNs + Laser; Group 5: CP‐HA MNs; Group 6: CP‐HA MNs + Laser; Group 7: BP‐CP‐HA MNs; Group 8: BP‐CP‐HA MNs + Laser. Data are presented as mean ± SD (*n* = 5), *p < 0.05, **p < 0.01, and ***p < 0.001.

On day 12, a variety of histological analyses were conducted to gain a deeper understanding of the wound healing process, focusing mainly on the newly formed epidermis, collagen deposition and angiogenesis. Hematoxylin and eosin (H&E) staining images revealed significantly increased epidermis thickness and lower inflammatory cells in BP‐CP‐HA MNs + Laser group (**Figure** **7**a,b), indicating the laser‐assisted MN system could not only alleviate the inflammatory response in the local microenvironment but also exhibit a speedy wound repair. Masson's section staining demonstrated the BP‐CP‐HA MNs + Laser group formed more collagen fiber deposition than any other groups (Figure 7c,d). Besides, CD31 immunohistochemical staining revealed the densest microvascular structures were generated in the wound site of BP‐CP‐HA MNs + Laser group (Figure 7e,f). Taken together, more collagen fiber deposition could induce a higher‐quality repair of wound tissue, and angiogenesis promoted the supplement of blood to the wound tissue, these favorable results in BP‐CP‐HA MNs + Laser group facilitated the process of wound healing.

### 2.7 Therapeutic Mechanism of BP‐CP‐HA MNs

**Figure 8:**
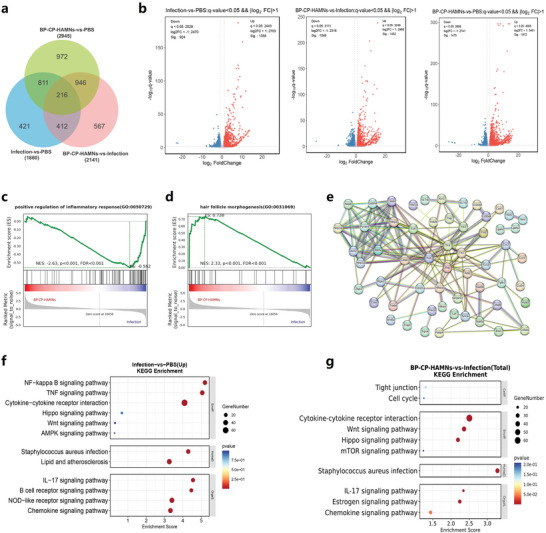
Therapeutic mechanism of BP‐CP‐HA MNs. a) Venn diagram showing the differentially expressed genes between different groups. b) Volcano plots showing the down‐regulated and up‐regulated genes between different groups. GSEA enrichment plots of GO (gene ontology) showing the c) down‐regulated “positive regulation of inflammatory response” pathway and d) up‐regulated “hair follicle morphogenesis” pathway after the treatment of BP‐CP‐HA MNs. e) PPI network analysis. KEGG enrichment analysis of differentially expressed genes in f) Infection‐versus‐PBS and g) BP‐CP‐HA MNs‐versus‐Infection.

To better reveal the potential mechanism of infectious wound healing in mice, RNA was enriched from mouse wounds treated with or without BP‐CP‐HA MNs and performed transcriptome sequencing. The transcriptome analysis initially focused on 6946 differentially expressed genes (DEGs) (**Figure** **8**a). Among these, 421 genes exhibited differential transcription only in the MRSA infection group, and 567 genes showed differential transcription only in the BP‐CP‐HA MNs treatment group. Furthermore, compared to normal mouse skin, 1358 genes were upregulated (indicated by red dots), and 924 genes were downregulated (indicated by blue dots) in MRSA infection group. Moreover, compared to the MRSA infection group, mouse skin treated with BP‐CP‐HA MNs showed upregulation of 1482 genes and downregulation of 1049 genes (Figure 8b).

Furthermore, Gene Set Enrichment Analysis (GSEA) revealed that biological functions positively regulating inflammatory response and hair follicle morphogenesis were significantly enriched in the BP‐CP‐HA MNs treatment group (Figure 8c,d). In addition, DNA replication, immune response, acute‐phase reaction, positive regulation of IL‐6 production and the canonical Wnt signaling pathway were significantly enriched in the BP‐CP‐HA MNs treatment group (Figure S36, Supporting Information). Previous studies have demonstrated that these signaling pathways are intricately linked to the suppression of inflammation in infected wounds and the regeneration of hair follicles,^[^ ^54^, ^55^ ^]^ thereby offering insights into the potential mechanisms by which BP‐CP‐HA MNs contribute to the repair and remodeling of infected wounds. A protein interaction (PPI) network was further established to reveal the interaction relationships between multiple inflammation related protein factors (Figure 8e). The Kyoto Encyclopedia of Genes and Genomes (KEGG) analysis revealed that the signaling pathway of cytokine‐cytokine receptor interaction, staphylococcus aureus infection, chemokine signaling and NF‐kappa B signaling pathway were significantly activated in MRSA‐infected mice (Figure 8f). After treatment with BP‐CP‐HA MNs, multiple inflammatory signaling pathways and staphylococcus aureus infection pathways were significantly inhibited, while cell cycle, tight junctions, and signaling pathways regulating pluripotency of stem cells were activated (Figure 8g). Therefore, the antibacterial and repair process might be proposed as follows: with laser irradiation, BP‐CP‐HA MNs could generate a profusion of ROS (^1^O_2_ and •OH) to kill MRSA for preventing bacterial infection. Then, with the improved degradation from toxic BP NSs to nontoxic phosphate and the biological role of copper ions, cell proliferation, migration and vascularization were induced to promote tissue regeneration. Besides, the improved biodegradation of toxic BP NSs also increased the biocompatibility of the MN system to relieve inflammation of the wound. In a word, these findings demonstrated that under the laser irradiation, BP‐CP‐HA MNs accelerated the wound healing process through preventing bacterial infection, inhibiting inflammation, promoting cell proliferation, and inducing the regeneration of hair follicles.

## 3 Conclusions

To combat the great challenges in administration limitation for bacterial pathogens‐infected wound healing, we designed and fabricated a multifunctional BP‐CP‐HA MN patch to realize the smart regulation of nanodynamic sterilization and tissue regeneration for efficient wound management. Thereinto, the matrix material HA could maintain the hard needle structure and the activity of nanomaterials. After penetrating the wound area, BP NSs and CP NDs could be released and exhibited versatile properties, such as enhanced photodynamic and chemodynamic activities, along with the gradual production of cell proliferation‐required phosphate from degraded BP NSs, enabling short‐term sterilization and long‐term tissue regeneration, effectively promoting MRSA biofilm‐infected wound healing. Both in vitro and in vivo systematic evaluations confirmed the excellent biocompatibility of BP‐CP‐HA MN system, along with its remarkable nanodynamic therapy and tissue regeneration capability. Additionally, DFT‐based MD calculations were conducted to reveal the improved BP degradation was triggered by Cu^2+^ and increased O_2_ enrichment. RNA‐seq technology was performed to reveal the potentially intrinsic therapeutic mechanism with different gene expression patterns and related signaling pathway changes. Such a versatile BP‐CP‐HA MN system provides a creative strategy for anti‐infection therapy, and may further promote the research progress of nanomaterials and MNs in biomedicine.

## 4 Experimental Section

### Materials

Polydimethylsiloxane microneedle (MN) molds were obtained from Shiling Laike Die Business Co., In (Guangzhou, China). Sodium hyaluronate (HA, Mw: 20–40 kDa) was purchased from Freda Biochem Co., Ltd (Shandong, China). Bulk black phosphorus was purchased from XFNANO Materials Tech Co., Ltd (Nanjing, China). N‐methylpyrrolidone (NMP), Polyvinylpyrrolidone (PVP, Mw: 45000–58000, K30), and copper oxide nanoparticles (CuO) were purchased from Adamas‐beta Chemical Reagent Co., Ltd (Shanghai, China). Sodium hydroxide (NaOH), hydrogen peroxide (H_2_O_2_, 30%), sulfuric acid (H_2_SO_4_) and potassium permanganate (KMnO_4_) were purchased from Sinopharm Chemical Reagent Co., Ltd (Shanghai, China). Copper chloride dihydrate (CuCl_2_⋅2H_2_O), 3,3′,5,5′‐tetramethylbenzidine (TMB), and 1,3‐Diphenylisobenzofuran (DPBF) were purchased from Aladdin Biochemical Technology Co., Ltd (Shanghai, China).

### Synthesis and Characterization of BP NSs

Bulk BP was dispersed in NMP and treated with probe sonication. Then BP NSs were obtained by centrifugation (6000 rpm, 10 min) to remove the non‐exfoliated bulk BP. Afterwards, TEM, DLS, UV‐Vis‐NIR spectra, AFM (atomic force microscope), XRD, Raman spectra were employed to reveal the properties of BP NSs, which further confirms the rationality of the synthesis route.

### Synthesis and Characterization of CP NDs

PVP (4 g) was firstly dissolved in 40 mL aqueous solution containing CuCl_2_∙2H_2_O (68.2 mg). Then, 40 mL NaOH (0.02 M) and 800 µL H_2_O_2_ were added sequentially. After stirring 30 min, the PVP‐coated CP NDs were collected by high‐speed centrifugation and washed with water for several times. Afterwards, TEM, UV‐Vis‐NIR spectra, XPS, XRD, KMnO_4_‐based colorimetric analysis were employed to reveal the properties of CP NDs, which further confirms the rationality of the synthesis route.

### Detection of ROS (•OH and ^1^O_2_) and O_2_ Generation

As for CP NDs, PBS buffer (pH 5.6 or 7.4) containing TMB (2 mM) was mixed with CP NDs (1 mM), then the increased absorption at 650 nm confirmed the pH‐responsive generation of ∙OH. By contrast, the TMB solution treated with Cu^2+^ (1 mM) or H_2_O_2_ (1 mM) alone was used as a control group. In addition, an oxygen meter (DO‐957, Shanghai Yidian Scientific Instruments Co., Ltd.) was used to detect the O_2_ generation within 10 min (pH 5.6 or 7.4). As for ^1^O_2_ production of BP NSs, 4 mL BP NSs solution (8 µg mL^−1^) was mixed with DPBF (25 µg mL^−1^) and CP NDs (0 or 0.12 mM), then the absorption at 426 nm was tested after 650 nm laser irradiation (0.668 W cm^−2^) for 0, 5, 10, 15, 20, 25 min.

### Regulation of BP NSs’ Degradation Behavior by CP NDs

BP NSs solution was mixed with CP NDs (0, 0.1, 0.2, 0.4, 0.8 mmol g^−1^ BP NSs) for several days, the color and absorption (Vis‐NIR by a spectrometer, or optical density at 630 nm by an enzyme‐labeled instrument) changes were recorded to identify the degradation behavior of BP NSs regulated by CP NDs.

### Fabrication and Characterization of BP‐CP‐HA MN Patch

All MN patches were fabricated by polydimethylsiloxane molds using a classic template replication method. Firstly, three types of paste: HA paste ([HA] = 150 mg mL^−1^), BP‐HA paste ([BP] = 30 mg mL^−1^, [HA] = 150 mg mL^−1^), CP‐HA paste ([CP] = 36 mM, [HA] = 150 mg mL^−1^) were prepared by dissolving corresponding materials in deionized water. Then the BP‐HA paste and CP‐HA paste were sequentially filled into the needle cavities via centrifugation (4000 rpm) and dried at 37 °C. Next, the HA paste was filled into the mold via centrifugation (4000 rpm) and dried at 37 °C to form the whole MN patch. Finally, the patch was carefully peeled from the mold to allow for further experiments. Macro camera, microscope, and SEM were used to show the morphology of MN patches. The dissolution process of BP‐CP‐HA MN patch was obtained by putting into the PBS buffer and piercing the porcine skin. Besides, 650 nm laser (0.668 W cm^−2^) was used to test the photothermal ability of MN patches.

### In Vitro Evaluation of Cytotoxicity, Cell Proliferation, Migration, and Vascularization

For cytotoxicity analysis, leaching solutions of HA MNs, BP‐HA MNs, CP‐HA MNs, and BP‐CP‐HA MNs with different dilution ratios were obtained by dissolving MNs of one patch in different volumes of DMEM (0.25, 0.5, 1, 2, 4, 8 and 16 mL). The highest concentration of leaching solution (dissolving MNs of one patch in 0.25 mL DMEM) was defined as 100% of leaching solution, so the concentration of the leaching solution was 1.56%, 3.13%, 6.25%, 12.5%, 25%, 50%, and 100% from low to high. Then the leaching solutions were co‐incubated with HUVECs for 24 h, and the cell viability was examined by CCK‐8 assay. The cells treated with MN leaching solution (50%) were stained with the live/dead staining kit, SYTO9‐Propidium Iodide (PI) for CLSM observation. In addition, MN leaching solution was co‐incubated with mouse erythrocytes to evaluate blood compatibility, distilled water and saline were used as positive and negative controls, respectively. The cell migration was evaluated by a cell scratch experiment, in which a straight scratch was created using a 200‐µL pipette tip. After co‐incubation with MN leaching solutions (50%) for 24 h, the scratch width and cell proliferation were observed. The vascularizing effect of MN leaching solutions (50%) on HUVECs cells was assessed by a tube formation experiment. After co‐incubation for 24 h, the number of tube nodes and tube length were recorded. What's more, the effects of phosphoric acid and laser irradiation (650 nm, 0.668 W cm^−2^) on cell proliferation were also evaluated.

### In Vitro Evaluation of Antibacterial and Antibiofilm Performances

MRSA was treated with ten different protocols: (1) PBS, (2) PBS + Laser, (3) HA MNs, (4) HA MNs + Laser, (5) BP‐HA MNs, (6) BP‐HA MNs + Laser, (7) CP‐HA MNs, (8) CP‐HA MNs + Laser, (9) BP‐CP‐HA MNs, (10) BP‐CP‐HA MNs + Laser, in which the MN leaching solutions were all 50%. The laser parameters in laser groups were all 650 nm, 0.668 W cm^−2^ for 5 min. After treatments, SYTO9‐PI staining kit and crystal violet were employed to assess the bactericidal and antibiofilm activity, the survival rate of MRSA was determined by plate counting. Besides, the treated bacteria were further co‐incubated with mouse erythrocytes to evaluate bacterial virulence. For SEM observation, MRSA of five laser‐treated groups were rinsed with PBS and fixed with 2.5% glutaraldehyde at 4 °C for 12 h. Sequentially, immobilized bacteria were dehydrated using a series of ethanol concentrations (30%, 50%, 70%, 90%, and 100%) for 10 min. After drying, the bacteria were observed by SEM with gold coating. For ROS measurement, MRSA of five laser‐treated groups were stained with DCFH‐DA (10 µM) for 30 min and inspected. A series of Assay Kits, including MDA Content Assay Kit, Respiratory Chain Complex Activity Assay Kits, and ATP Content Assay Kit were employed to measure the MDA level, bacterial respiratory chain complexes activities, and ATP content.

### In Vivo Therapeutic Assessment

The animal experiments were approved by the Experimental Animal Management and Ethics Committee of Shanghai Tenth People's Hospital (SHDSYY‐2024‐4840). Female BALB/c mice (5‐6 weeks) were used to evaluate the wound healing capability and process of the MN patches in vivo. The round wounds (≈1 cm) were constructed, infected by MRSA for one day and then randomly divided into the following eight groups (*n* = 5): (1): PBS; (2): HA MNs; (3): BP‐HA MNs; (4): BP‐HA MNs + Laser; (5): CP‐HA MNs; (6): CP‐HA MNs + Laser; (7): BP‐CP‐HA MNs; (8): BP‐CP‐HA MNs + Laser. All treatments were conducted every other day for a total of three times, and the laser parameters in laser groups were all 650 nm, 0.668 W cm^−2^ for 10 min. Wounds were photographed on days 0, 3, 6, 9, and 12, and the wound areas were calculated using ImageJ. On day 3, the bacterial colonies of the wound sites were counted on LB plates. On day 6, ROS levels of the wound sites were analyzed by dihydroethidium. On day 12, the wound tissues were treated with H&E, Masson, and CD31 immunohistochemistry staining for histological analysis. The main organs were observed by H&E staining. Blood samples were collected for blood routine examination and serum biochemistry examination.

### Transcriptome Analysis

The wound tissues from control, MRSA‐infected, and BP‐CP‐HA MNs‐treated mice were harvested on day 12 post‐treatment, and total RNA was extracted using standard protocols. The RNA concentration and quality were assessed using the RNA Nano 6000 Assay Kit on the Bioanalyzer 2100 system (Agilent Technologies, California, USA), following the manufacturer's guidelines. The RNA purification, reverse transcription, library construction, and sequencing were performed at OE Biotech Co., Ltd (Shanghai) using the Illumina Novaseq platform according to the instructions in the manual. The gene expression values in RNA‐seq were represented by FPKM (Fragments Per Kilobase of transcript per Million mapped reads) to correct for sequencing depth and gene length. Differential expression analysis was performed using the DESeq2 statistical software package in R (with a fold change ≥ 2 and q < 0.05).

### Statistical Analysis

Microsoft Excel 2021, Origin 2024, and IBM SPSS Statistics 27 were used for data statistics and statistical significance calculation. The test data were expressed as mean ± standard deviation (SD). The level of significance was analyzed on the basis of two‐tailed Student's *t*‐test. *p < 0.05, **p < 0.01, ***p < 0.001.

## Conflict of Interest

The authors declare no conflict of interest.

## Supporting information



Supporting Information

## Data Availability

The data that support the findings of this study are available from the corresponding author upon reasonable request.
