# Supplementary material for: Engineered Microneedle System Enables the Smart Regulation of Nanodynamic Sterilization and Tissue Regeneration for Wound Management
Source: Adv Sci (Weinh). 2025 Jan 13;12(9):2412226. doi: 10.1002/advs.202412226 (PMC11884594; doi:10.1002/advs.202412226)
Supplement: Supplementary file 1 — Supporting Information [file ADVS-12-2412226-s001.docx]

Supporting Information

**Engineered Microneedle System Enables the Smart Regulation of Nanodynamic** **Sterilization and Tissue Regeneration for** **Wound Management**

*Shiyang Lin,^#1^ Zhongqi Cui,^#1^ Qingqiong Luo,^#2^ Chen Li,^1^ Yue Zhang,^1^ Fengjiao Yang,^1^ Yichuan Chen,^3^ Chuansheng Xu,^1^ Yan Gao,^1^ Shasha Zhao,^1^ Fenyong Sun,^1^ Dandan Shen,*^1^ Qi Wu,*^4, 5^ and Shuo Shi*^1^*

^1^School of Chemical Science and Engineering, Department of Laboratory Medicine, Shanghai Tenth People's Hospital of Tongji University, Tongji University, Shanghai 200092, PR China.

^2^Department of Clinical Laboratory Medicine, Shanghai Skin Disease Hospital, School of Medicine, Tongji University, Shanghai 200443, PR China.

^3^Tongji University School of Medicine, Tongji University, Shanghai 200092, PR China.

^4^Department of Clinical Laboratory, Shanghai Children's Hospital, Shanghai Jiao Tong University School of Medicine, Shanghai 200062, PR China.

^5^Key Laboratory of Endemic and Ethnic Diseases, Ministry of Education, Guizhou Medical University, Guiyang 550004, PR China.

E-mail: shishuo@tongji.edu.cn (S. Shi), wuqi496@163.com (Q. Wu), sdd2832907@163.com (D. Shen).


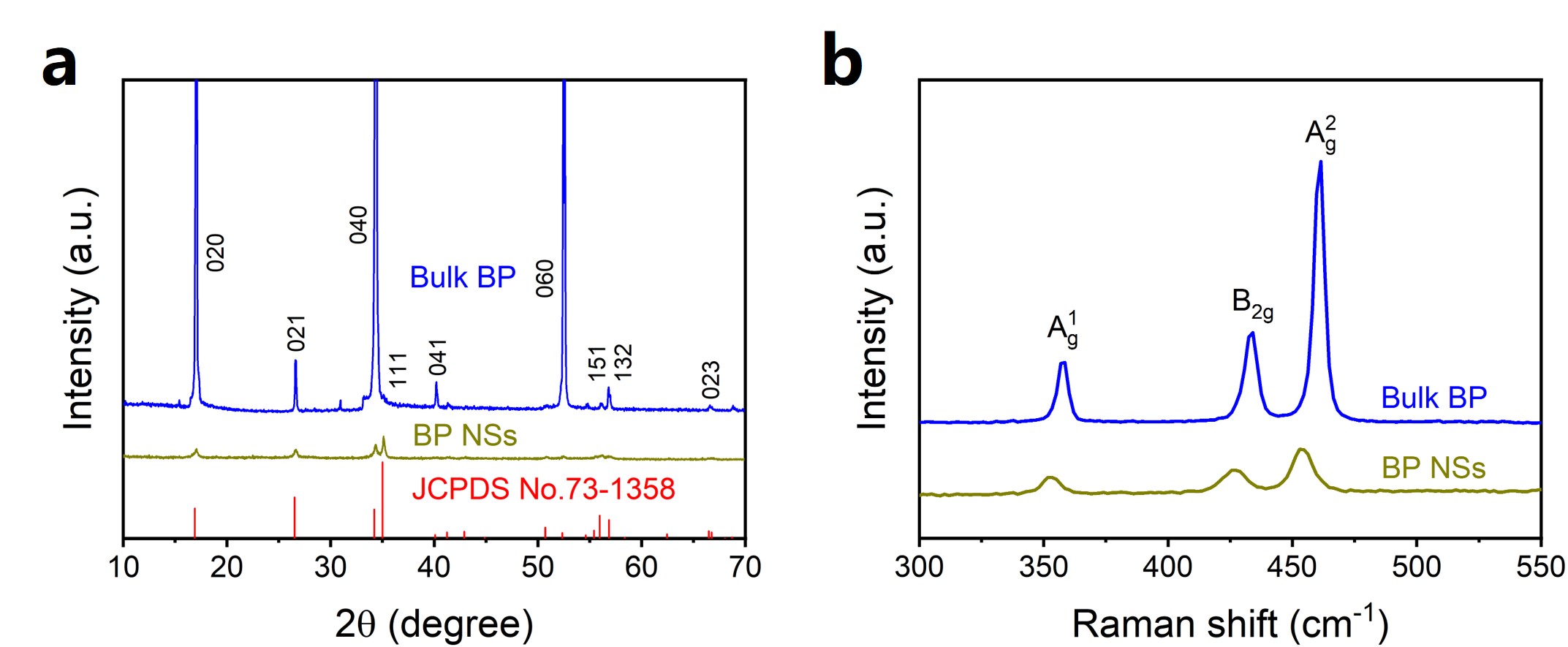


**Figure S1.** (a) XRD patterns, (b) Raman spectra of bulk BP and BP NSs.


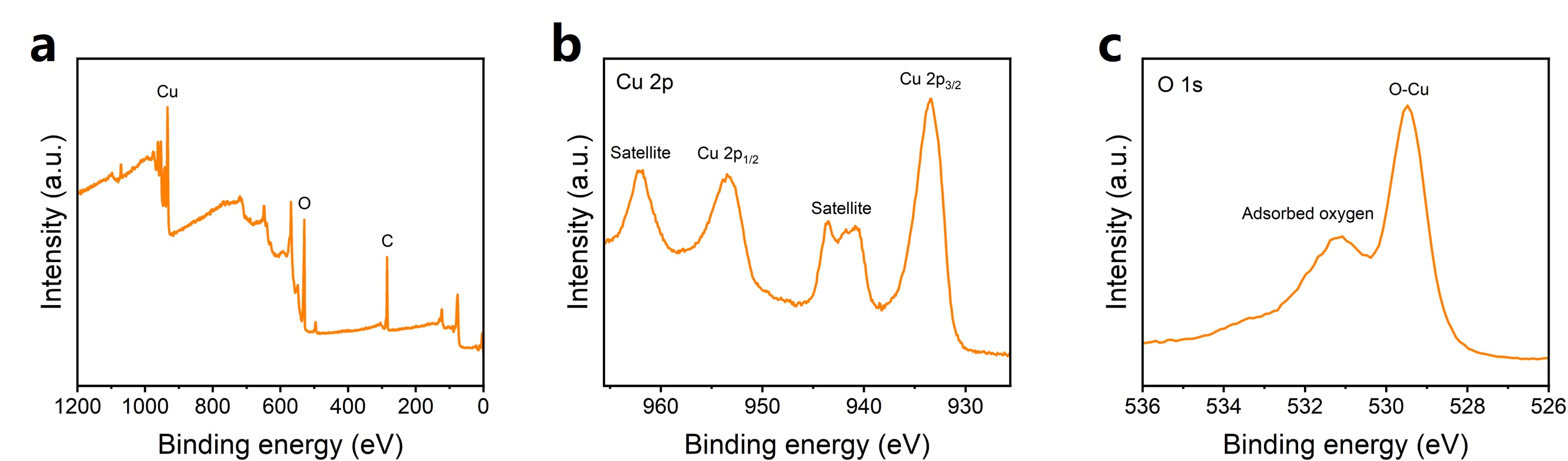


**Figure S2.** XPS spectra for (a) CuO, (b) Cu 2p, and (c) O 1s.


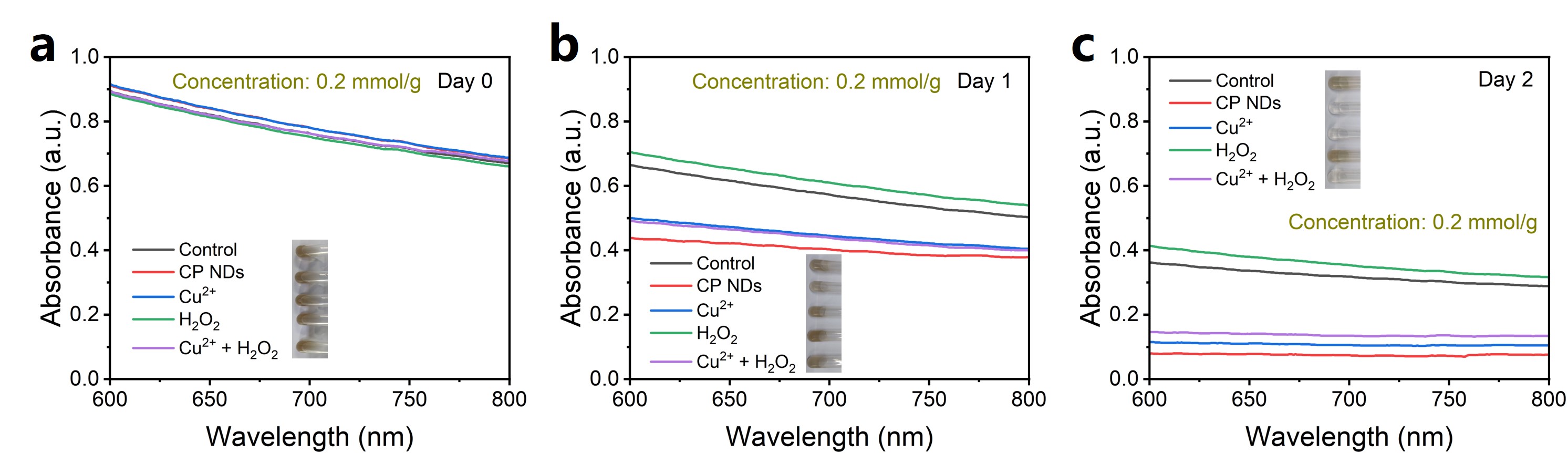


**Figure S3.** Absorption spectra and photographs (inset) of BP NSs incubating with H_2_O, CP NDs, Cu^2+^, H_2_O_2_, Cu^2+^ + H_2_O_2_ (0.2 mmol g^-1^ BP NSs) on (a) day 0, (b) day 1, (c) day 2.


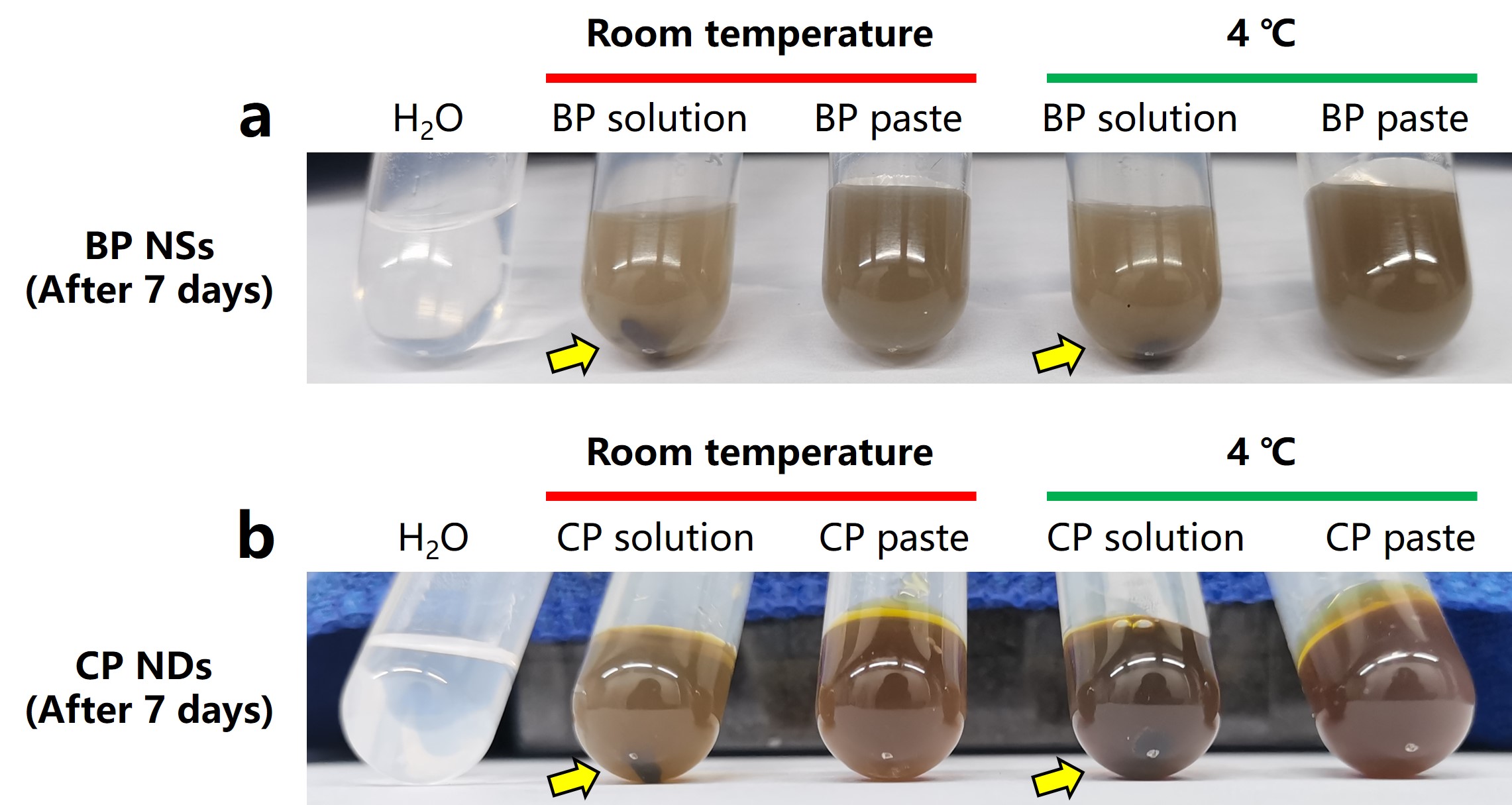


**Figure S4.** Photographs of (a) BP NSs and (b) CP NDs dispersed in deionized water and H_2_O dissolved HA paste after 7 days.


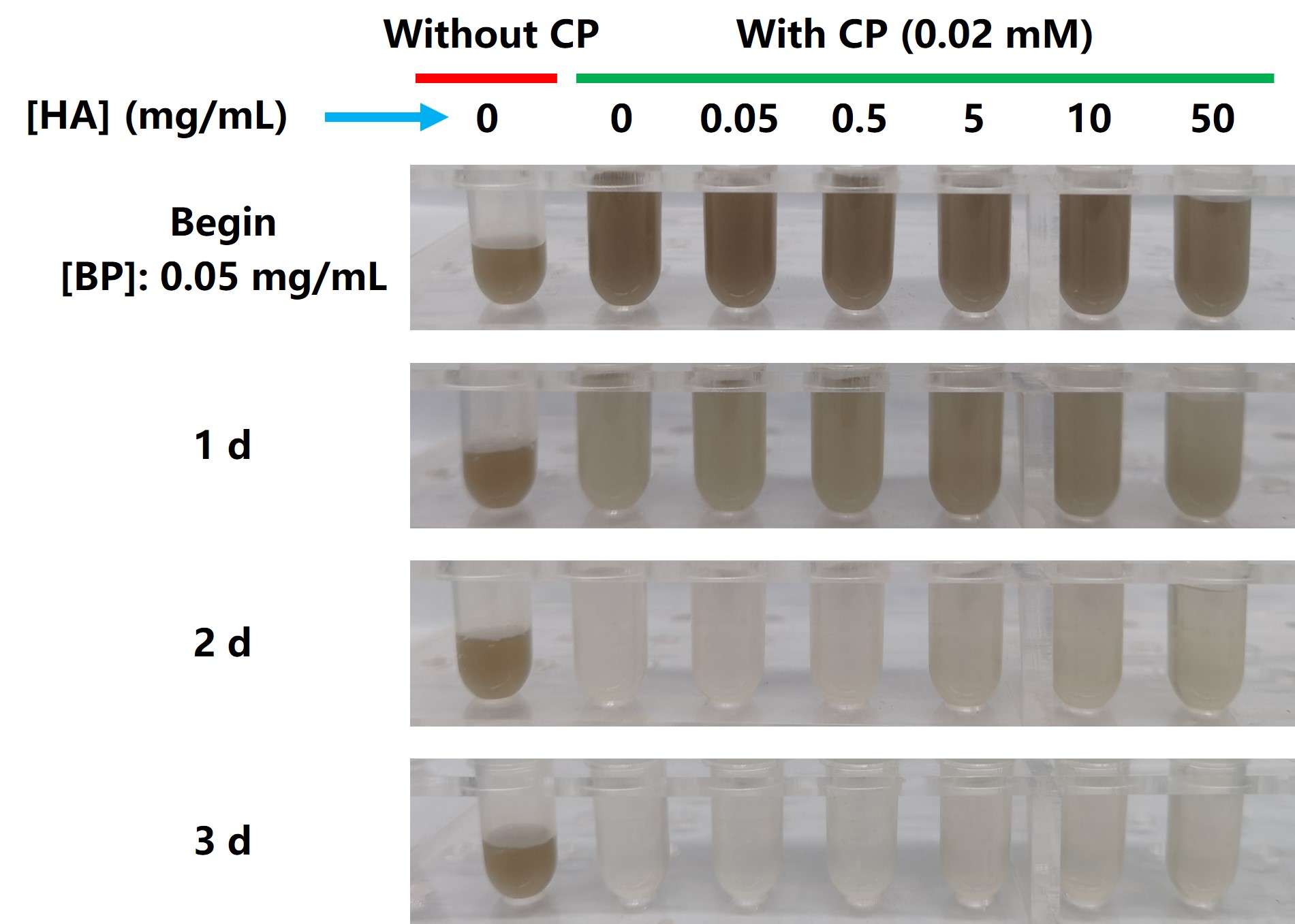


**Figure S5.** Photographs showing the degradation of BP NSs under different conditions.


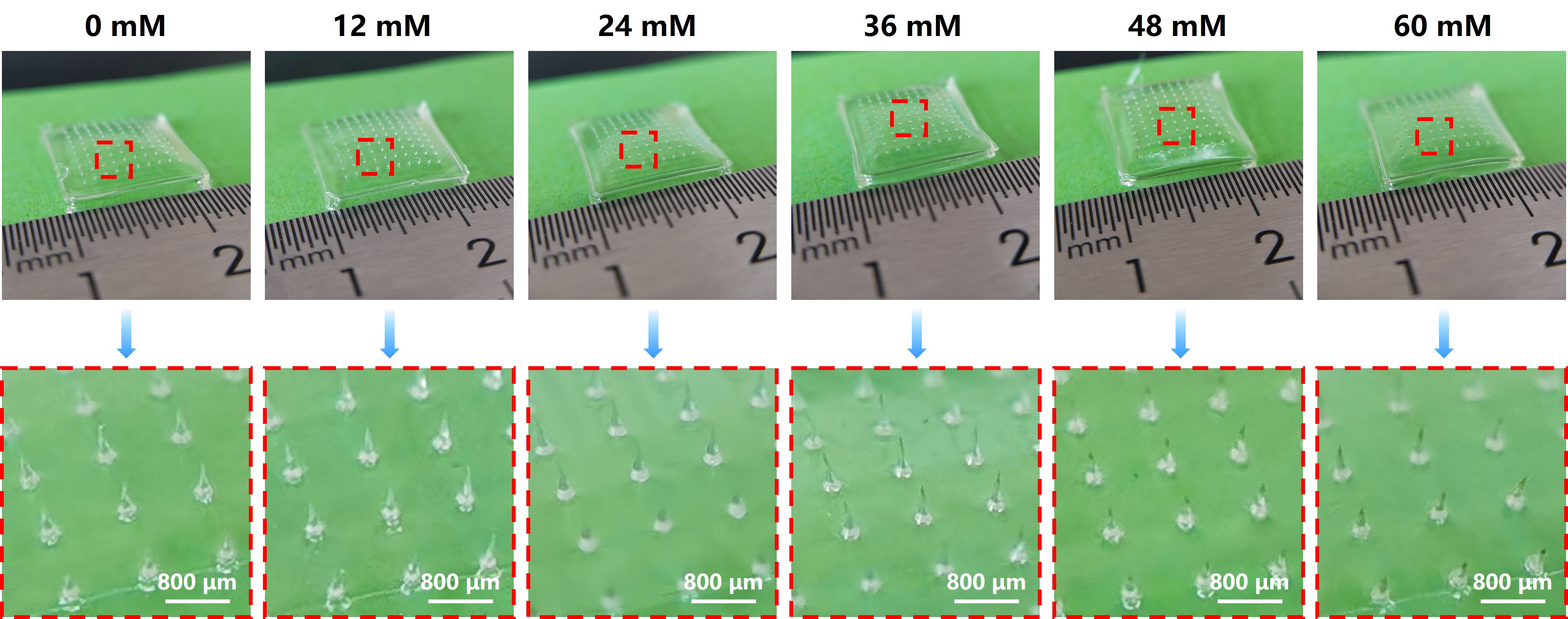


**Figure S6.** Photographs of CP-HA MN patches fabricated by different concentrations of CP-HA paste.


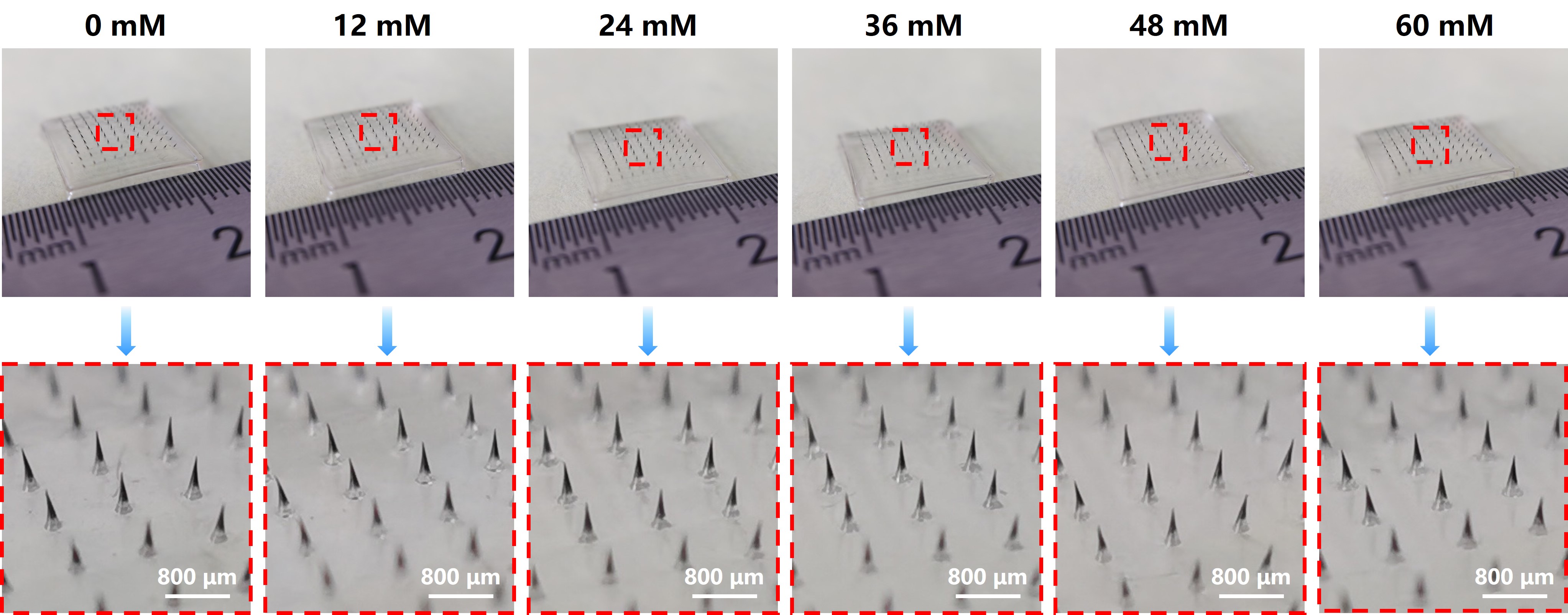


**Figure S7.** Photographs of BP-CP-HA MN patches fabricated by BP-HA paste (30 mg mL^-1^) and different concentrations of CP-HA paste.


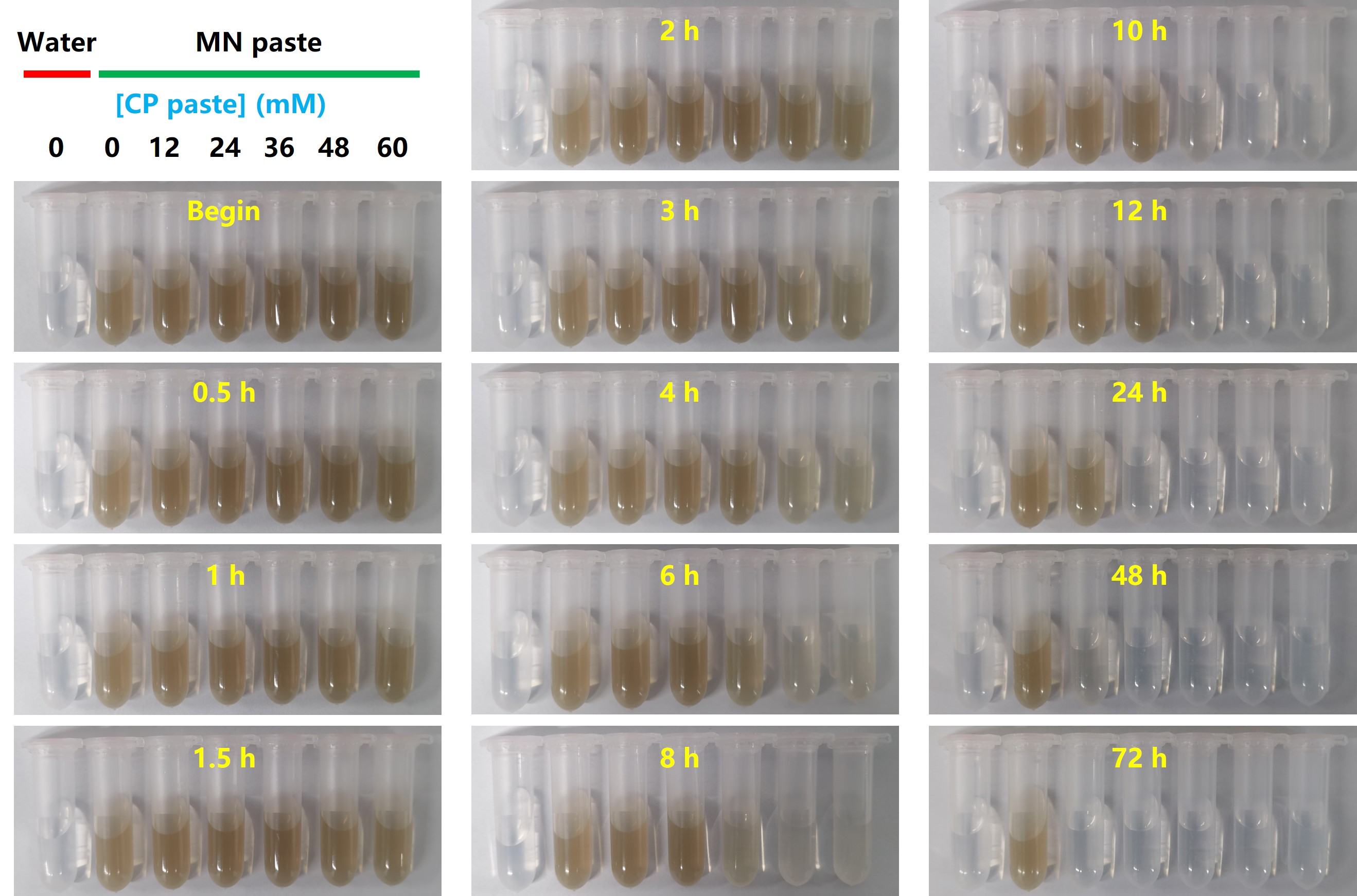


**Figure S8.** Photographs showing the degradation of BP-CP-HA MNs (with different concentrations of CP NDs). The controllable degradation of BP NSs could be easily achieved by adjusting the concentrations of CP-HA paste in the process of fabricating the MN patches.


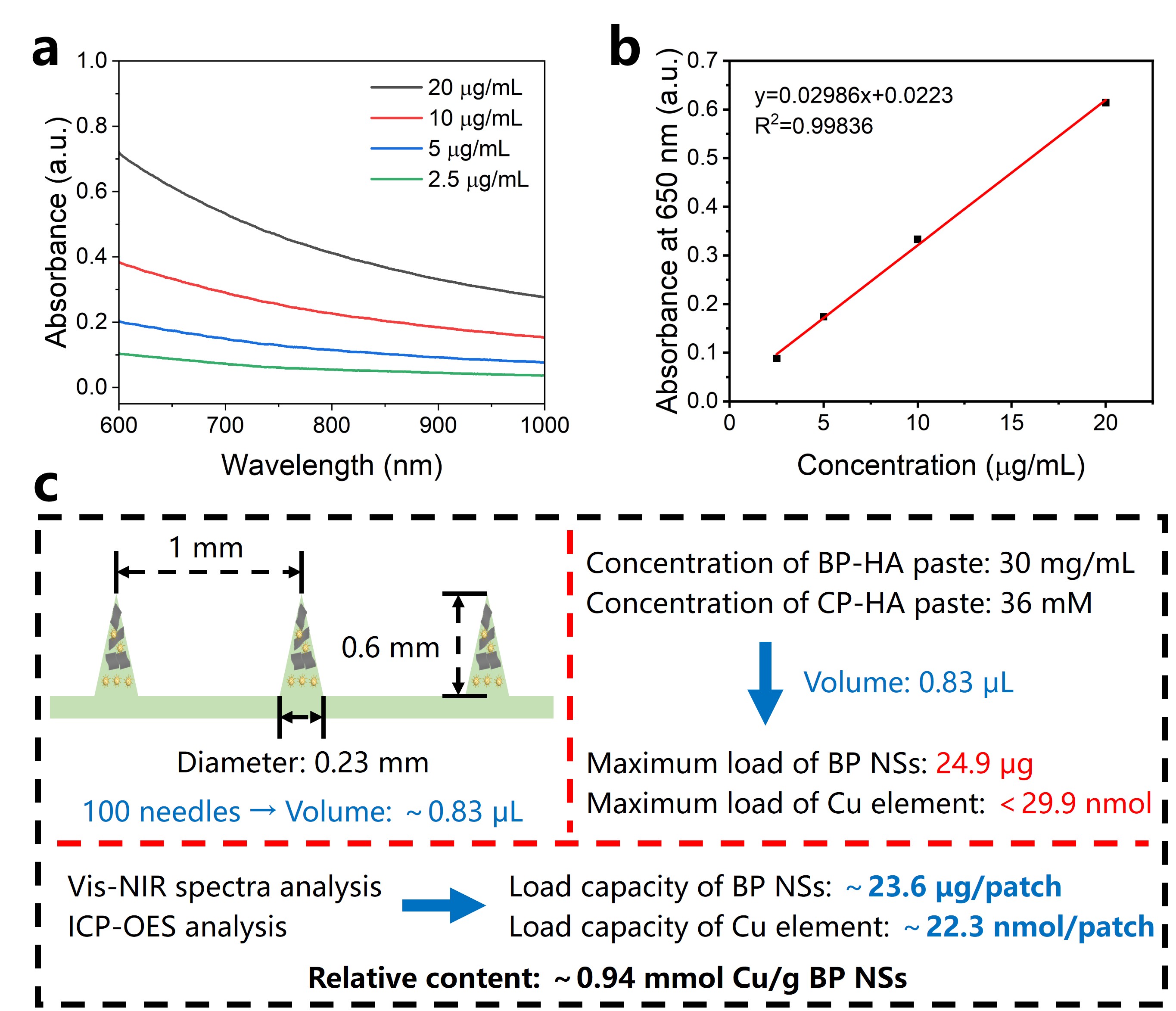


**Figure S9.** (a) Vis-NIR spectra of BP NSs and (b) the linear fitting at 650 nm. (c) Quantitative calculation of BP NSs and Cu^2+^.


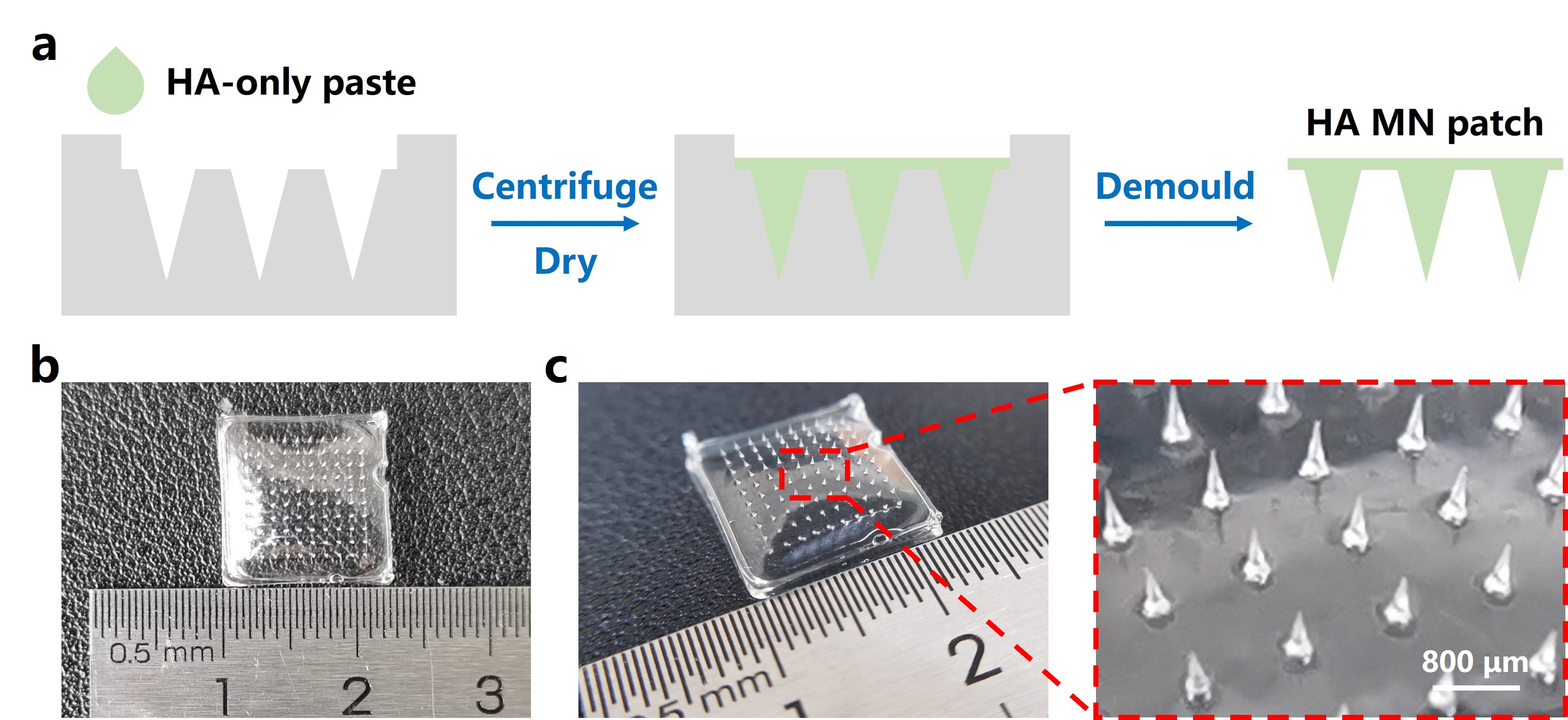


**Figure S10.** (a) Diagrammatic sketch of the fabrication of HA MN patch. Representative (b) photograph and (c) macro photograph of HA MN patch. The local magnified image showing the needle arrays.


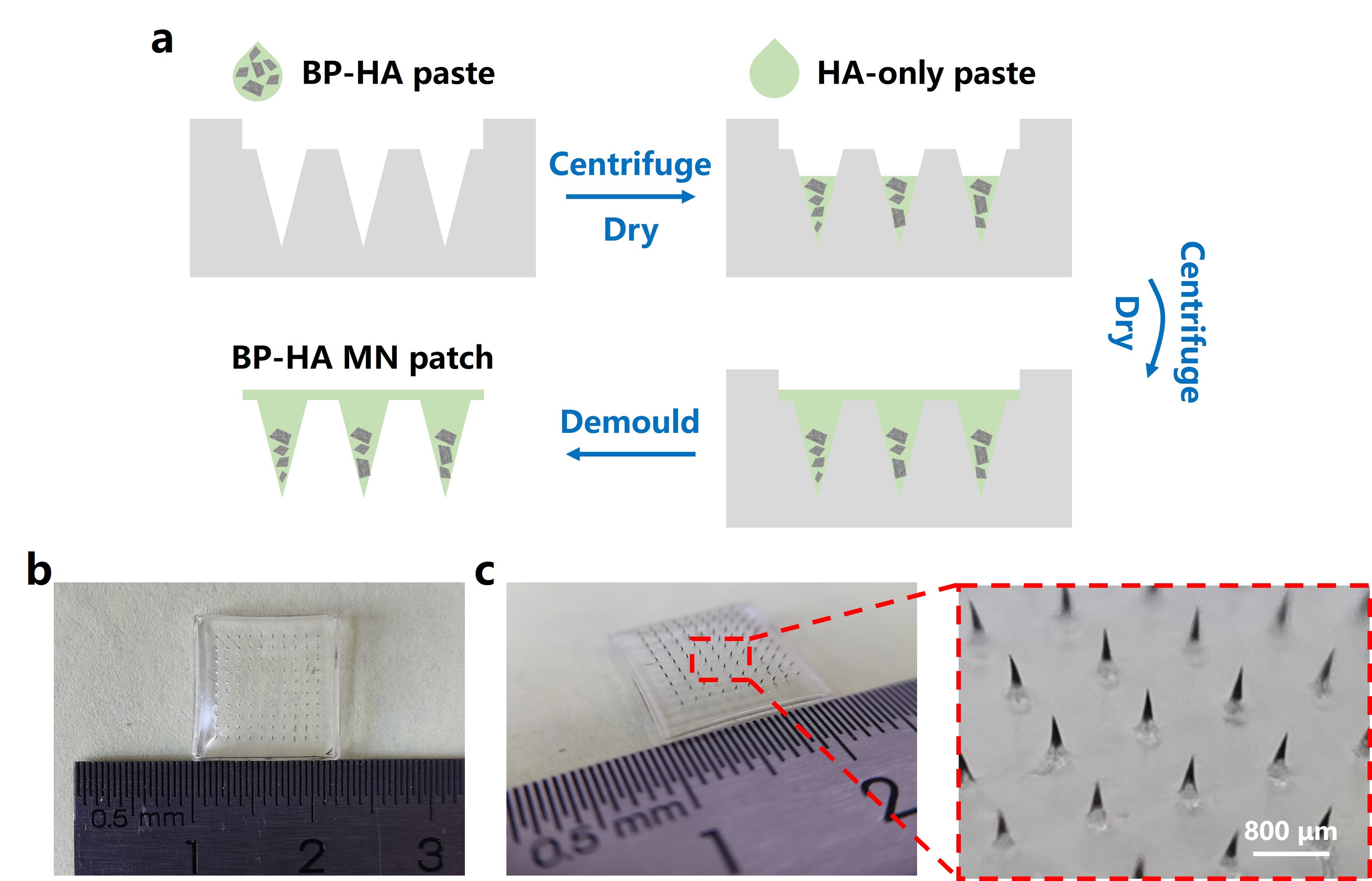


**Figure S11.** (a) Diagrammatic sketch of the fabrication of BP-HA MN patch. Representative (b) photograph and (c) macro photograph of BP-HA MN patch. The local magnified image showing the needle arrays.


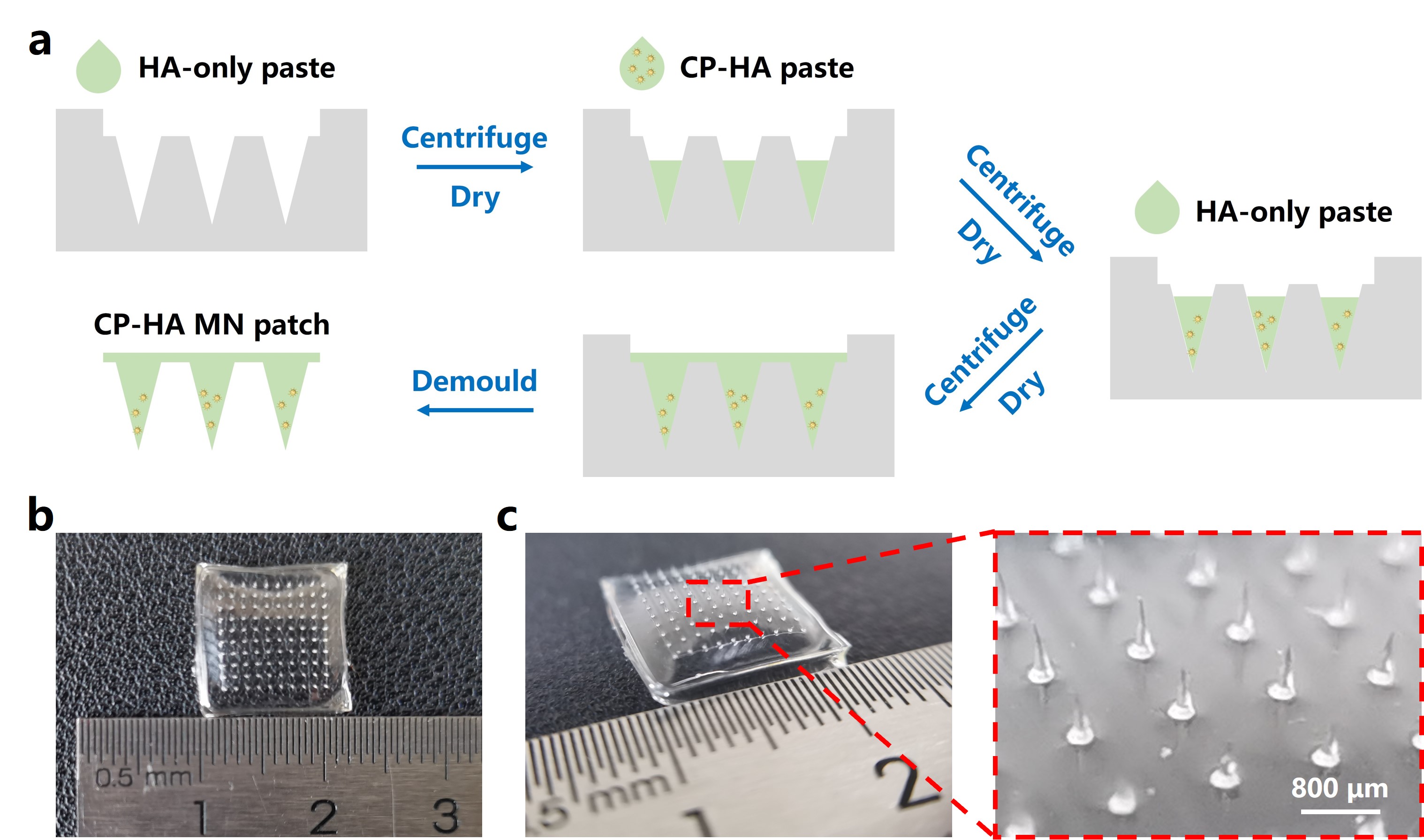


**Figure S12.** (a) Diagrammatic sketch of the fabrication of CP-HA MN patch. Representative (b) photograph and (c) macro photograph of CP-HA MN patch. The local magnified image showing the needle arrays.


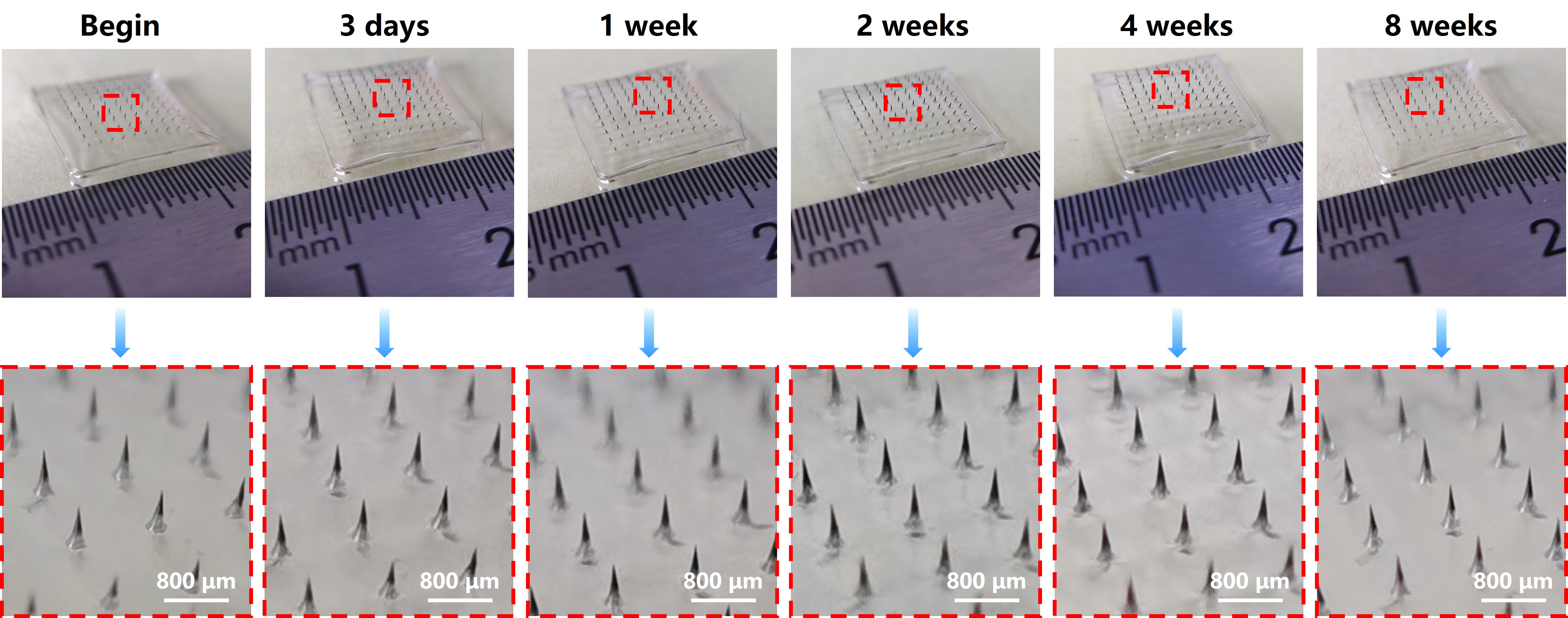


**Figure S13.** Photographs of a BP-CP-HA MN patch at different time points.


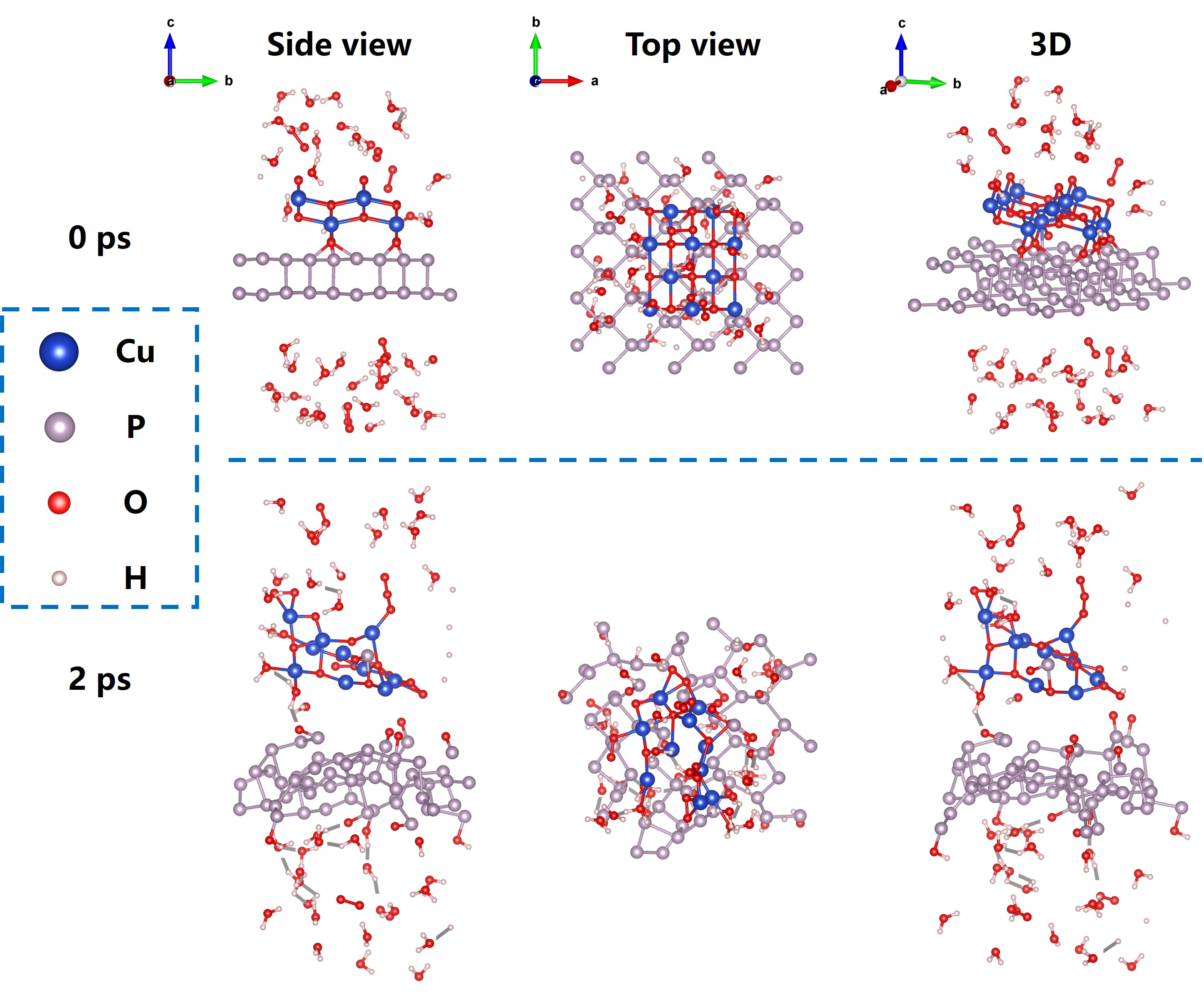


**Figure S14.** Structural evolution path of monolayer BP NSs and CP NDs in ambient system (310 K) contains H_2_O and O_2_.


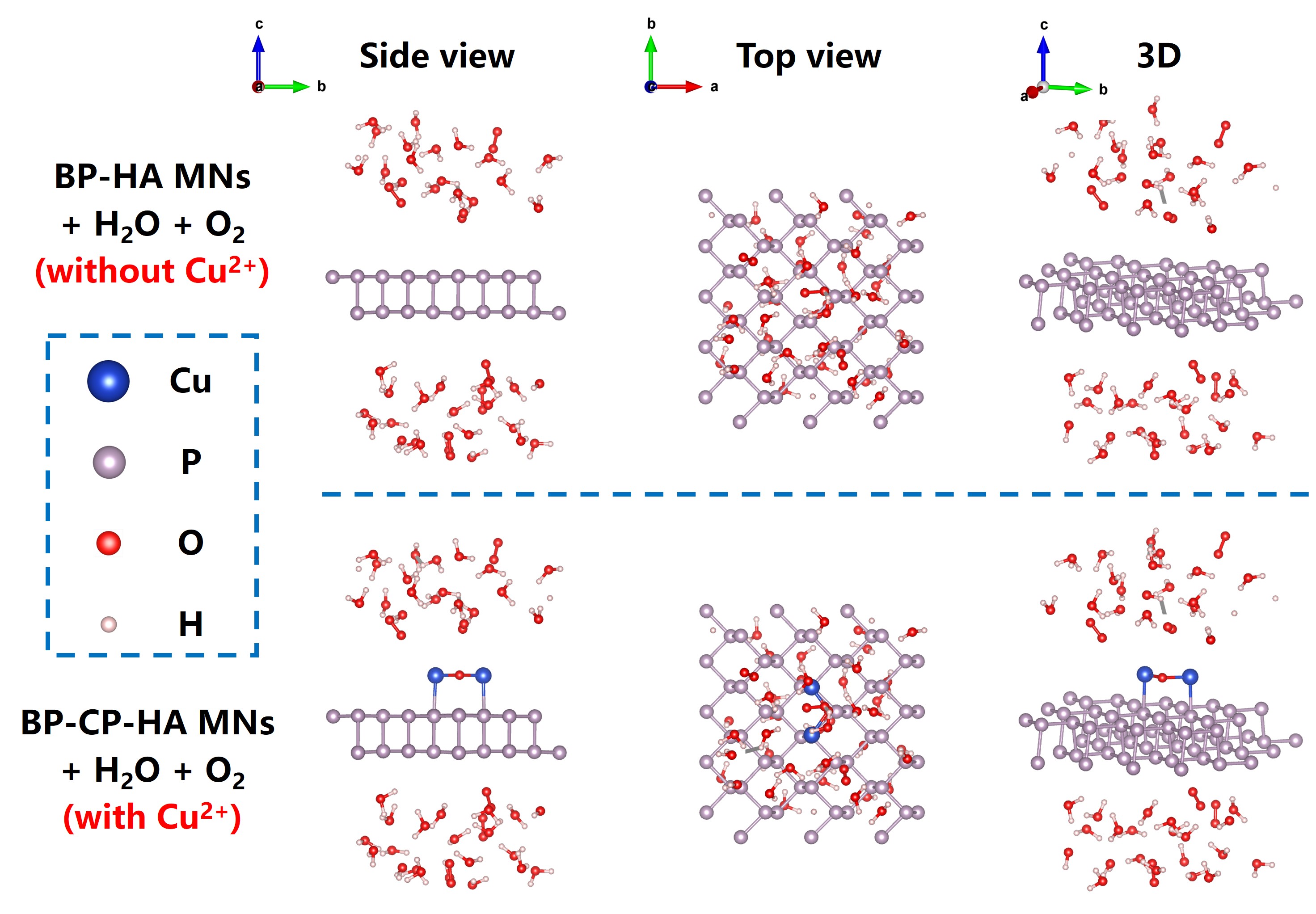


**Figure S15.** Density functional theory (DFT)-based molecular dynamic (MD) simulation models of monolayer BP NSs (with or without Cu^2+^) in ambient condition of H_2_O and O_2_ after the MNs are dissolved.


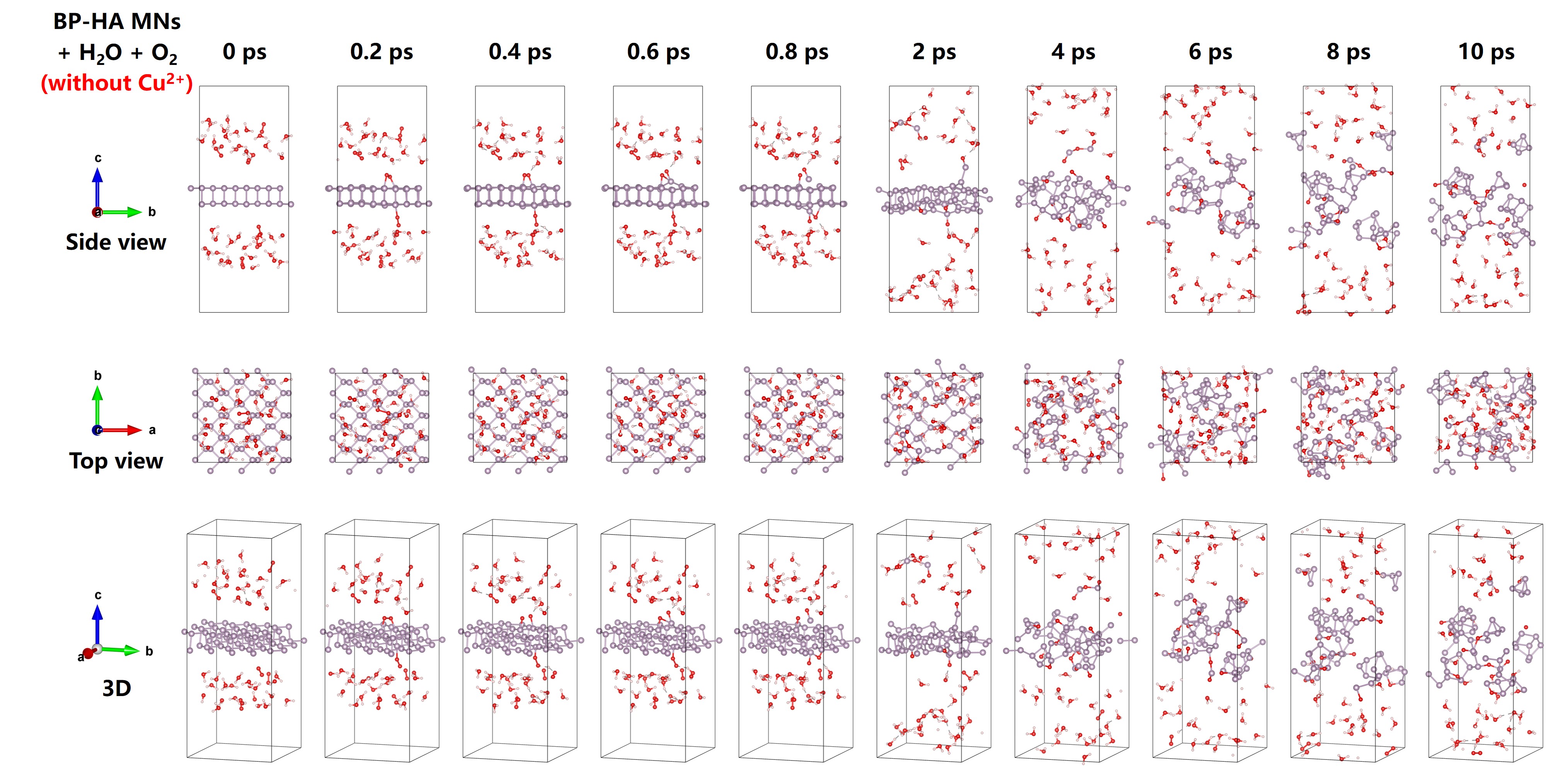


**Figure S16.** Structural evolution path of monolayer BP NSs in ambient system (310 K) contains H_2_O and O_2_.


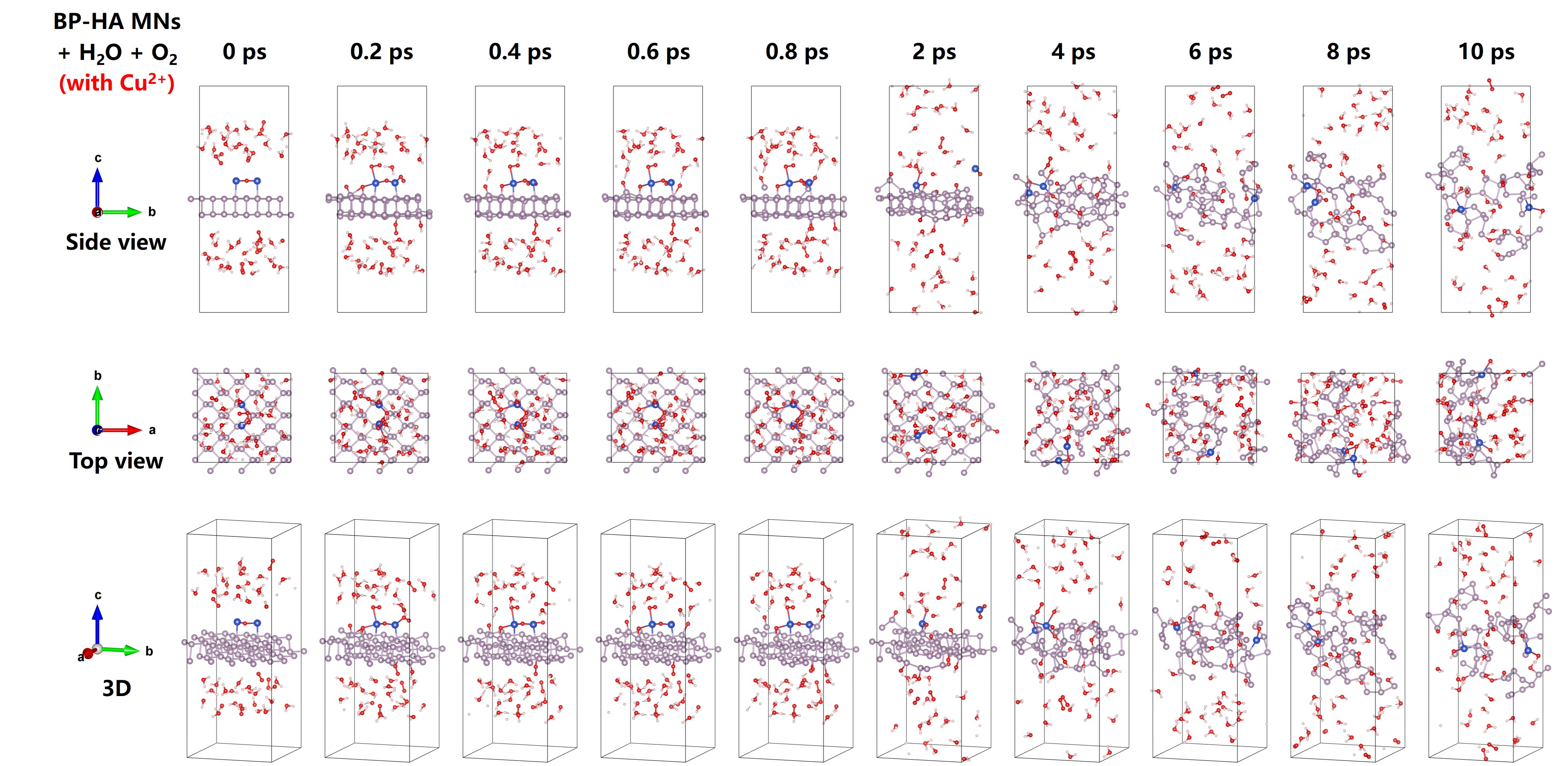


**Figure S17.** Structural evolution path of monolayer BP NSs in ambient system (310 K) contains H_2_O, O_2_ and Cu^2+^.


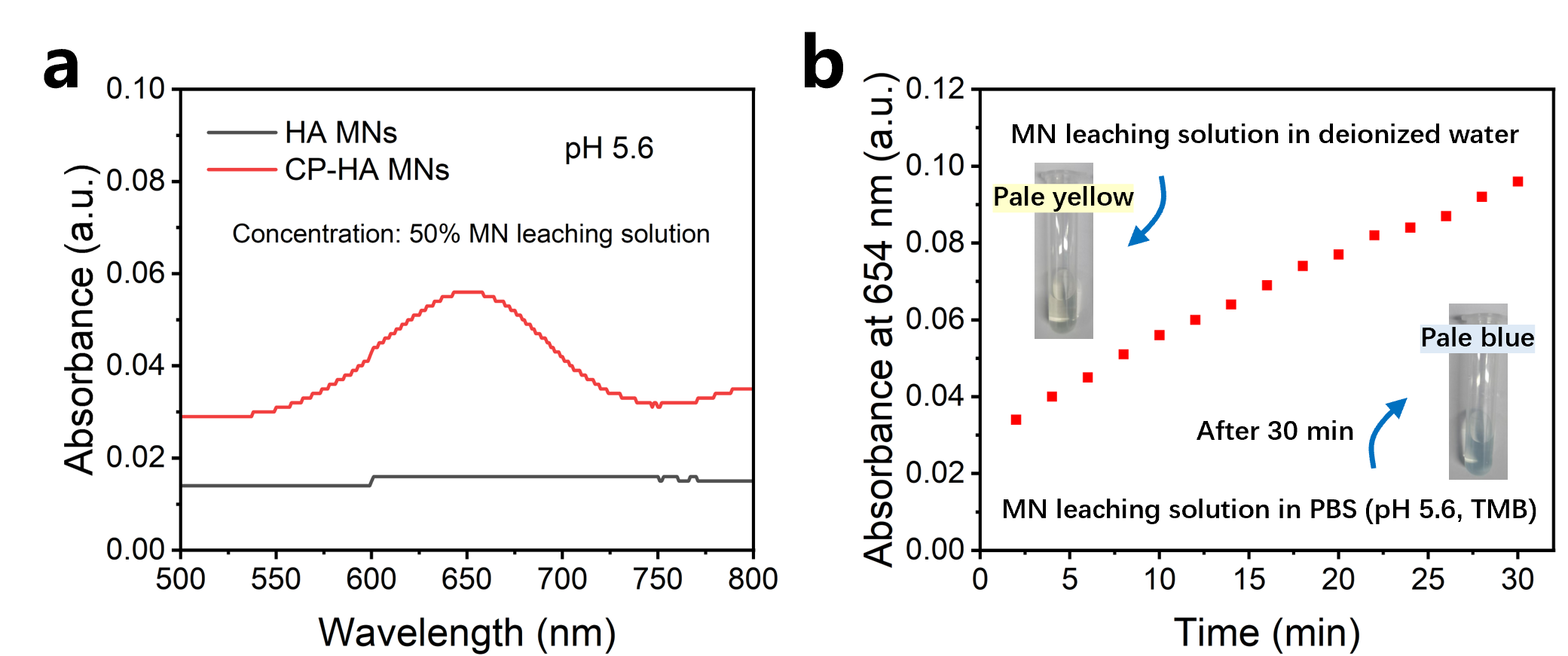


**Figure S18.** (a) Absorption spectra of TMB solution treated with 50% leaching solution of HA MNs and CP-HA MNs (pH = 5.6, 10 min). (b) Time-dependent absorption changes (654 nm) of TMB solution treated with 50% leaching solution of CP-HA MNs at pH 5.6. Even though the content of CP NDs in 50% CP-HA MNs leaching solution was low (pale yellow), color transformation could be found, indicating the production of •OH by Fenton-like reaction.


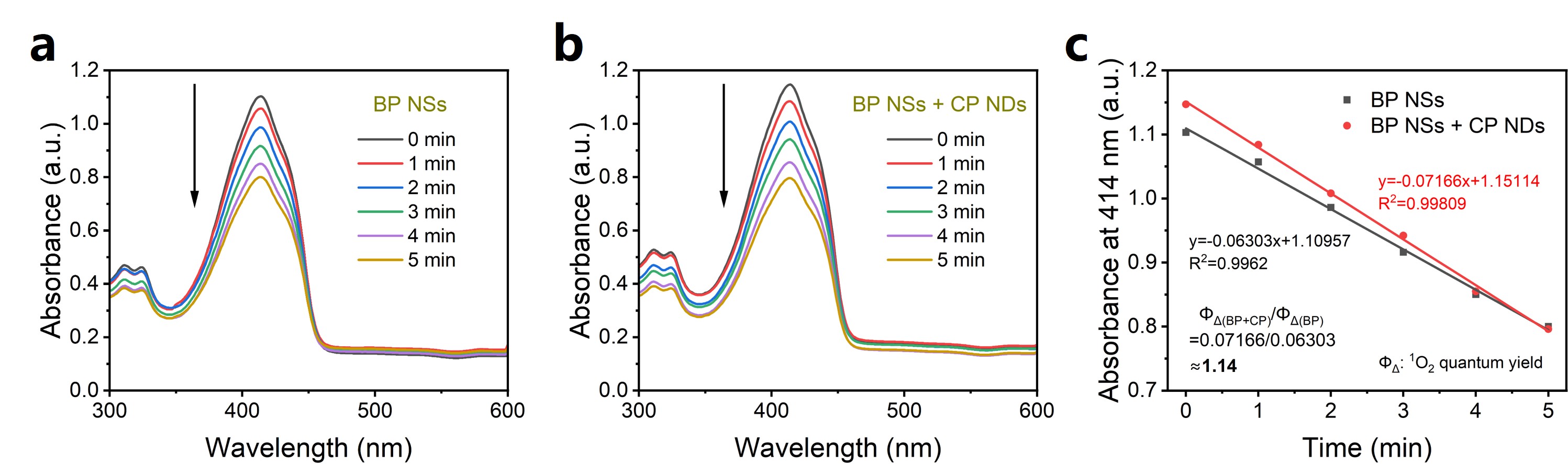


**Figure S19.** Time-dependent absorption spectra of DPBF treated with (a) BP NSs, (b) BP NSs + CP NDs in DMF under laser irradiation (650 nm, 0.668 W cm^-2^). Relative concentration: ~0.94 mmol CP NDs g^-1^ BP NSs. (c) Linear fitting of the degradation of DPBF.


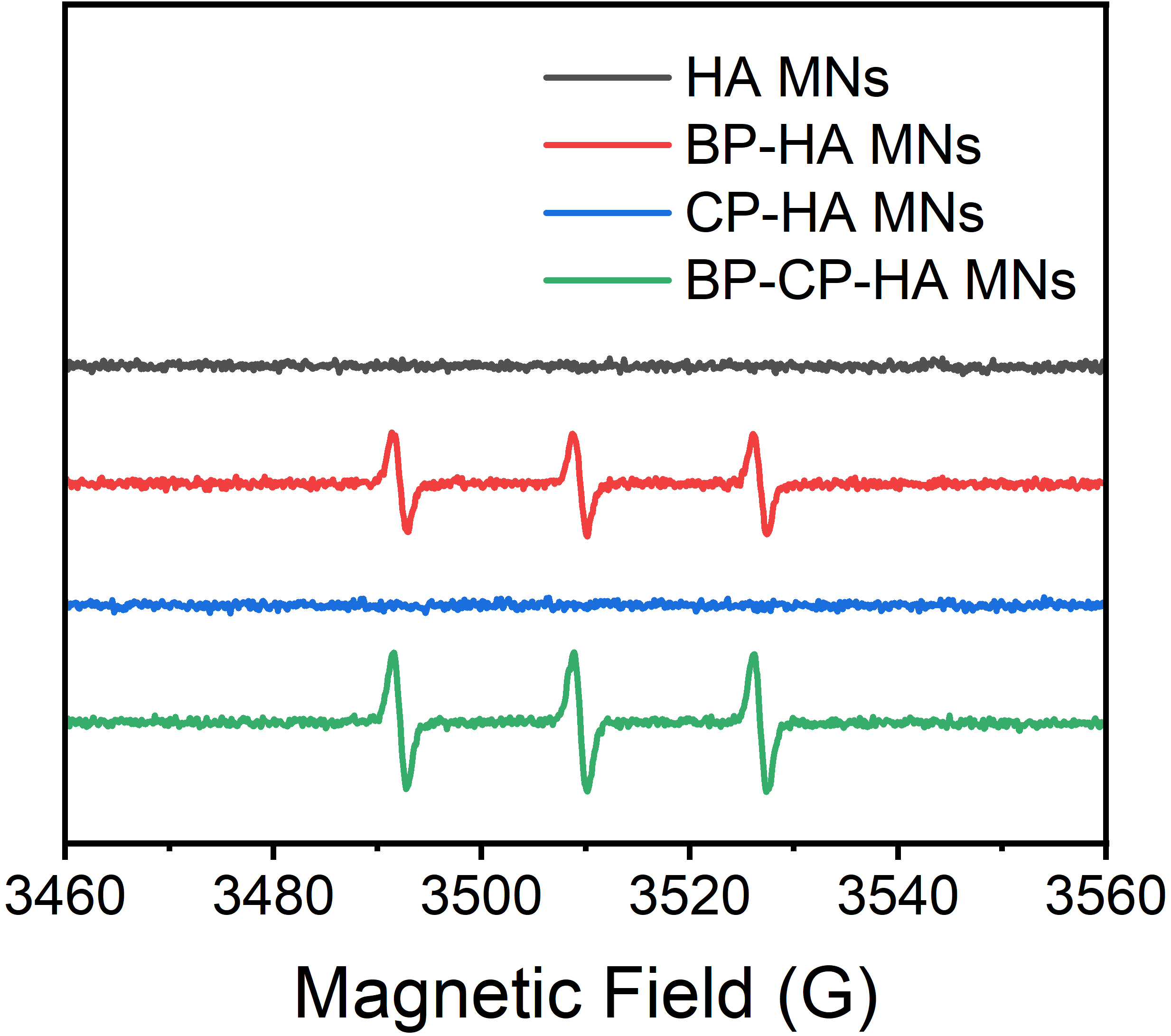


**Figure S20.** ESR spectra of TEMP/^1^O_2_ adducts in different solutions (leaching solutions of HA MNs, BP-HA MNs, CP-HA MNs and BP-CP-HA MNs in PBS, pH = 5.6), demonstrating the enhanced ^1^O_2_ generation ability of BP NSs by CP NDs.


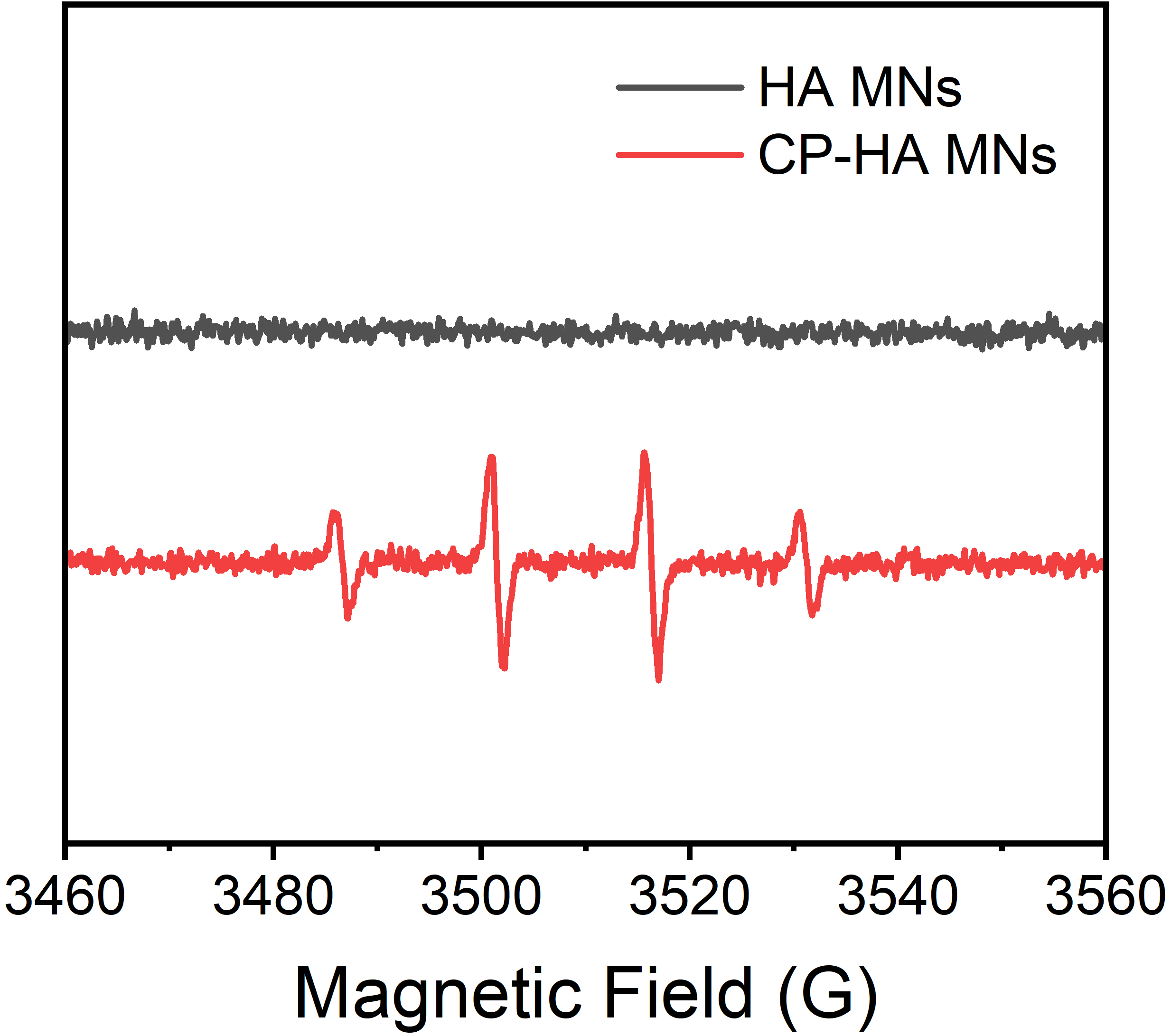


**Figure S21.** ESR spectra of DMPO/•OH adducts in different solutions (leaching solutions of HA MNs and CP-HA MNs in PBS, pH = 5.6), demonstrating the •OH generation ability of CP NDs.


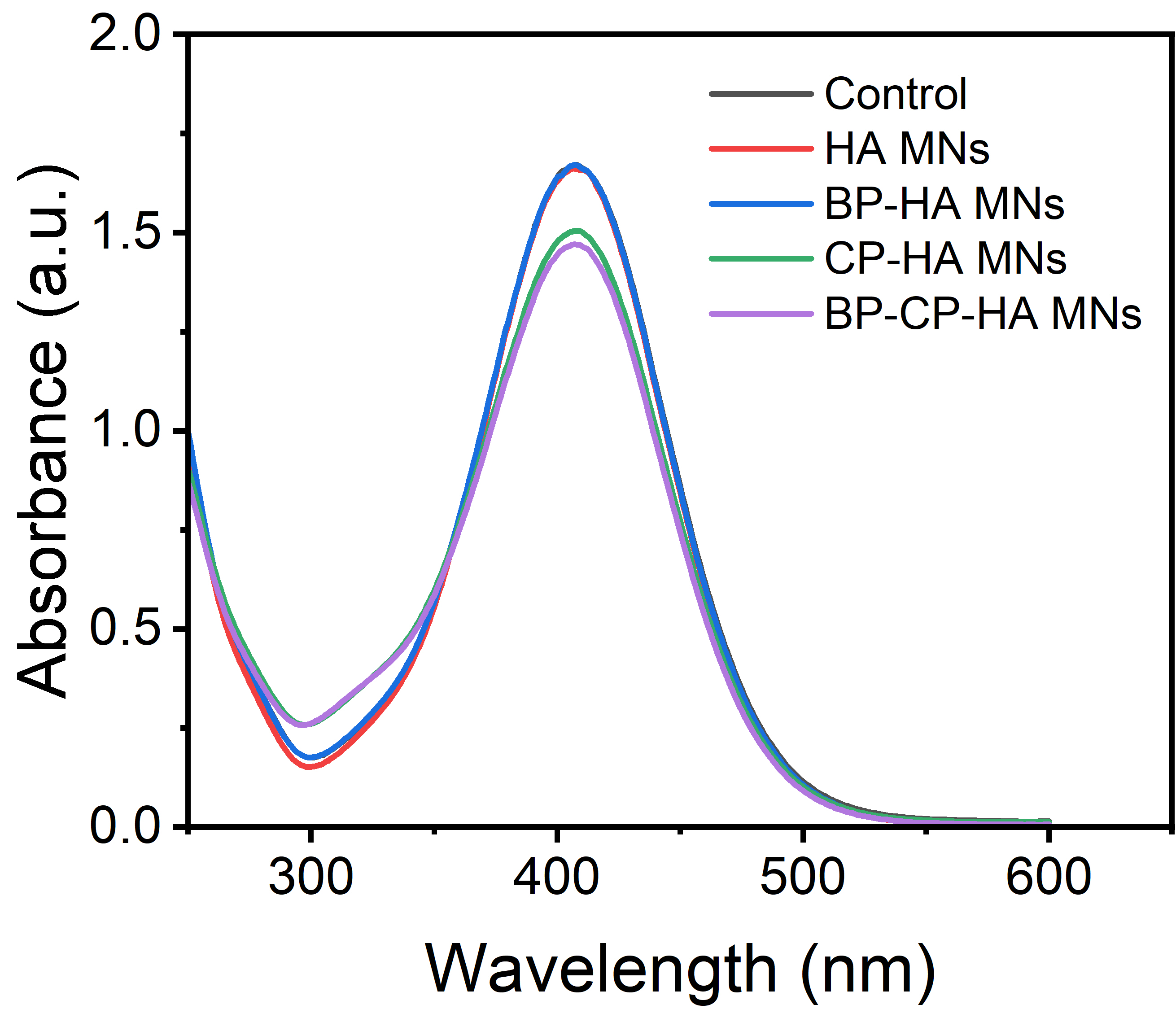


**Figure S22.** Absorption spectra of DTNB for the detection of GSH depletion. 1mL MN leaching solution (50%) was mixed with 20 μL GSH (0.2 mM) for 30 min. After centrifugation, the GSH content in the supernatant was evaluated using DTNB (0.1 mM). All materials were dissolved in Tris-HCl buffer solution (10 mM).


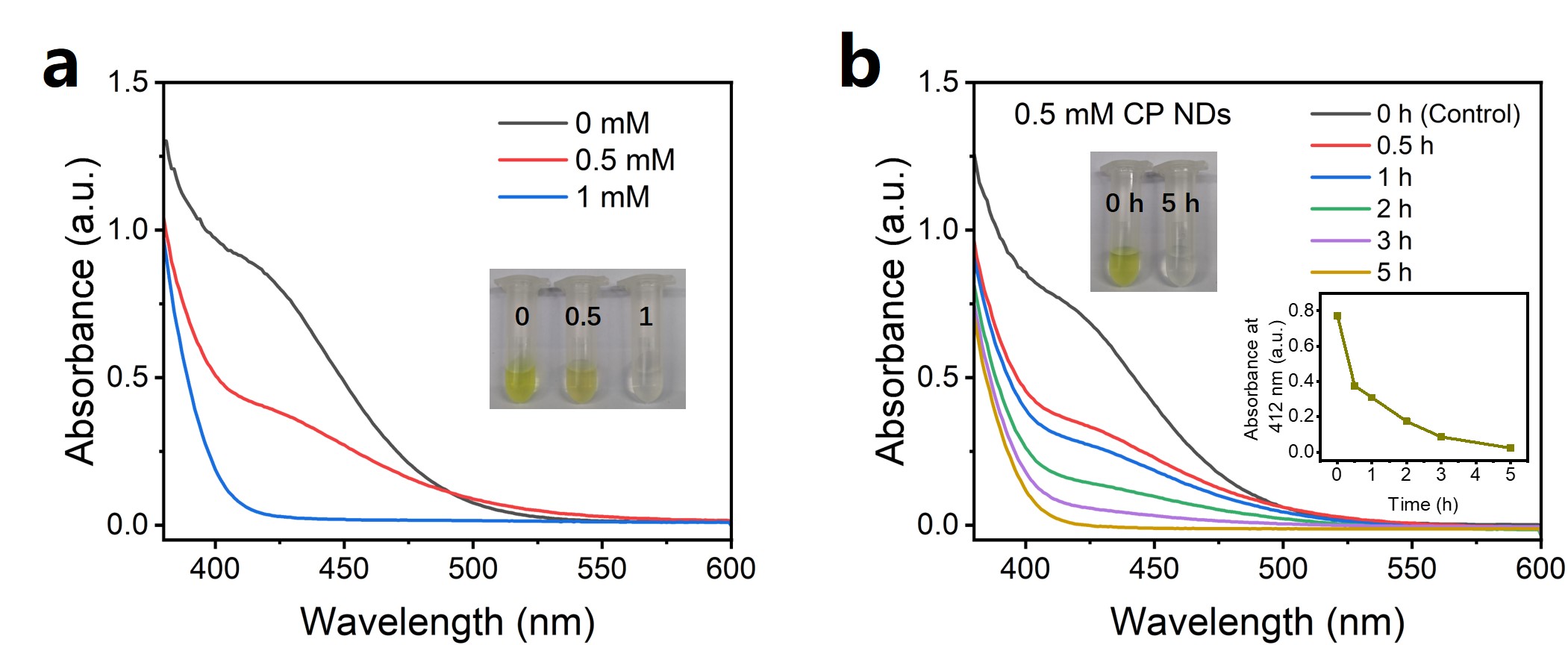


**Figure S23.** (a) CP NDs concentration-dependent absorption spectra and photographs (inset) of DTNB after 5 min of reaction between CP NDs and GSH. (b) CP NDs time-dependent absorption spectra and photographs (inset) of DTNB. Inset: the corresponding characteristic absorbance at 412 nm of GSH after treatment with 0.5 mM CP NDs. CP NDs, GSH (0.08 mM) and DTNB (0.2 mM) were dissolved in Tris-HCl buffer solution (10 mM).


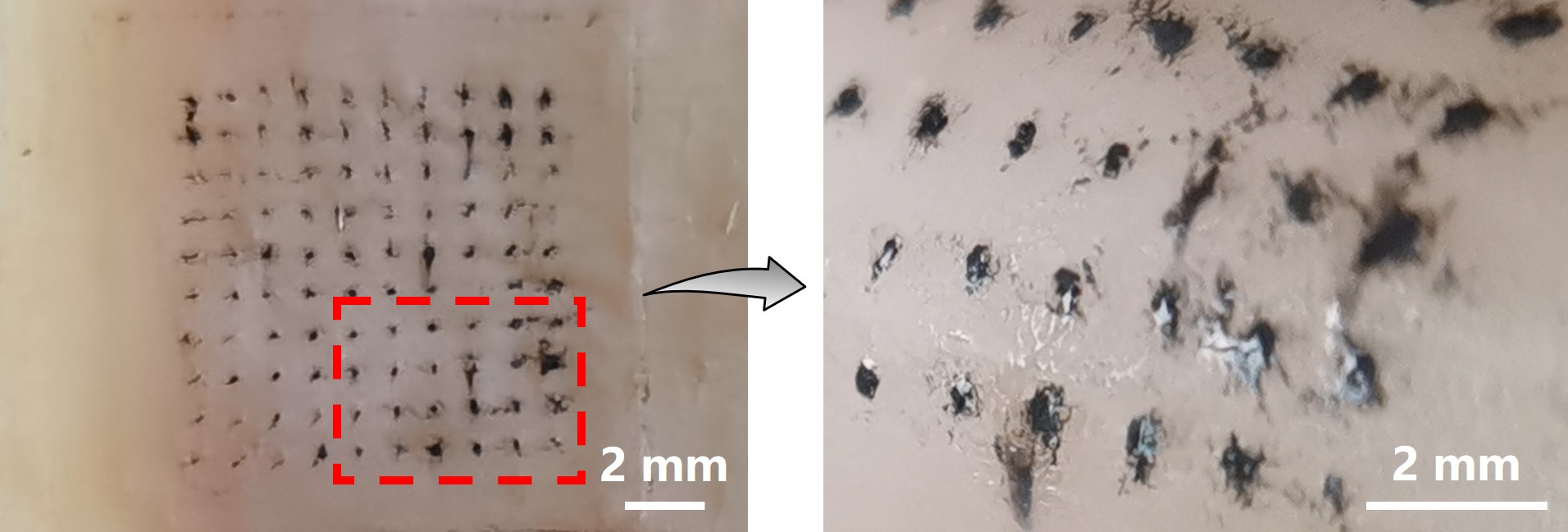


**Figure S24.** Porcine skin treated by a BP-CP-HA MN patch showing the microchannels filled with released materials.


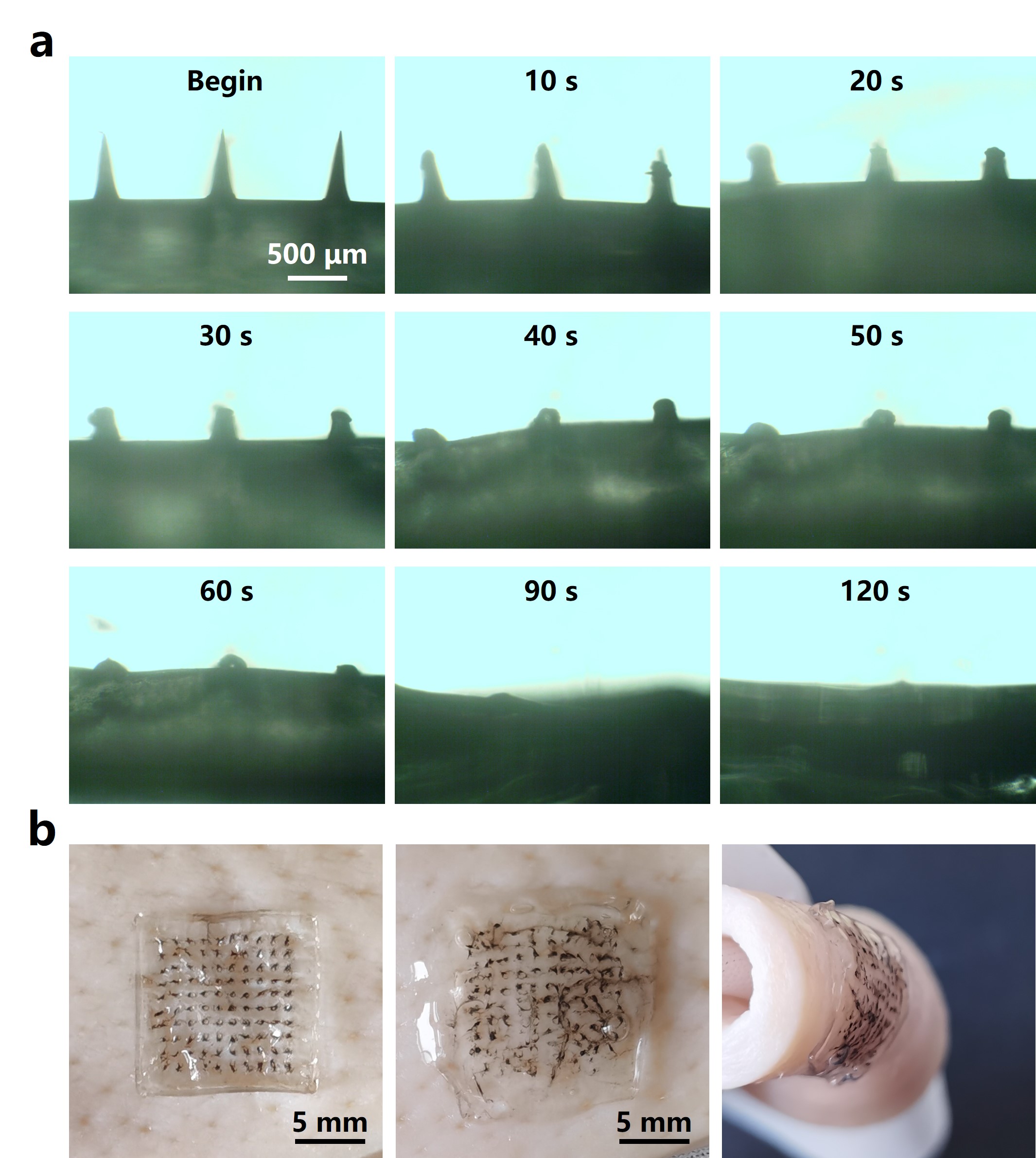


**Figure S25.** (a) Microscopic images of BP-CP-HA MNs after being inserted into porcine skin and dissolving for different times. (b) Photographs of a BP-CP-HA MN patch adhering to porcine skin.


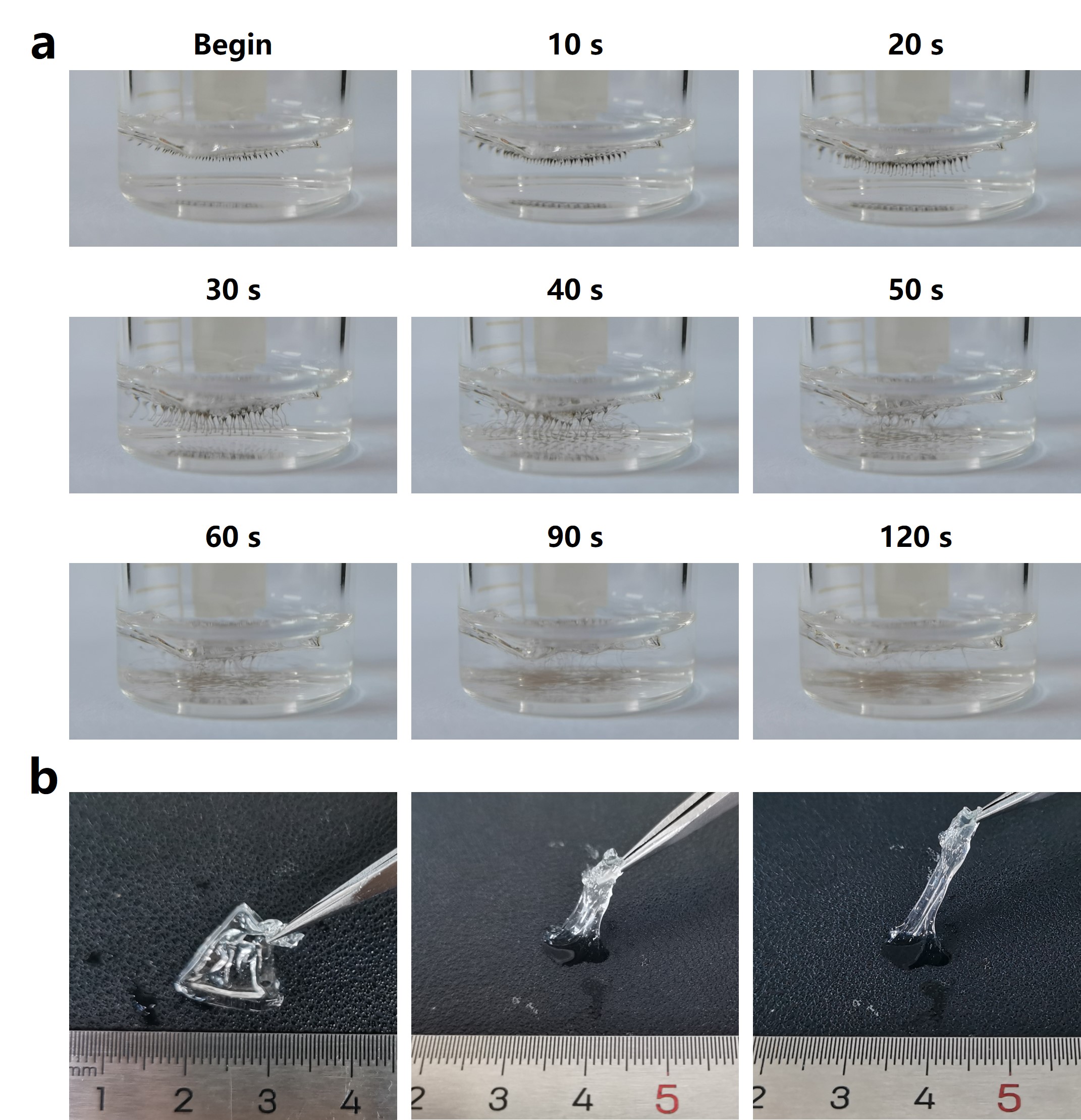


**Figure S26.** (a) Dissolution photographs of a BP-CP-HA MN patch in PBS (pH = 7.4) with prolonged durations. (b) Photographs of the back layer showing the adhesion of MN patch.


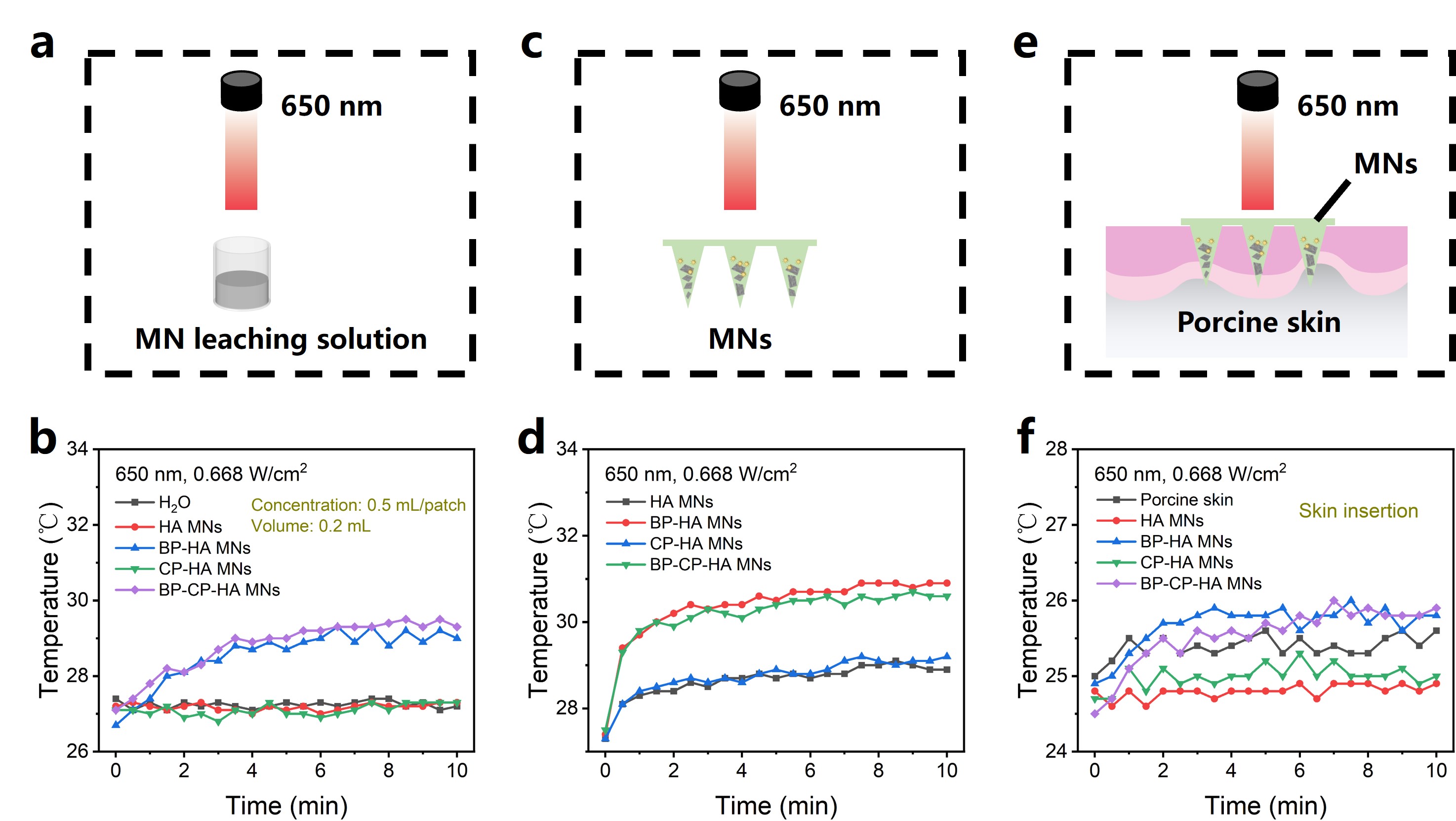


**Figure S27.** (a) Schematic diagram of the photothermal experiment using the MN leaching solution and (b) the corresponding temperature changes. (c) Schematic diagram of the photothermal experiment of four MN patches and (d) the corresponding temperature changes. (e) Schematic diagram of the photothermal experiment of four MN patches after penetration into the porcine skin and (f) the corresponding temperature changes.


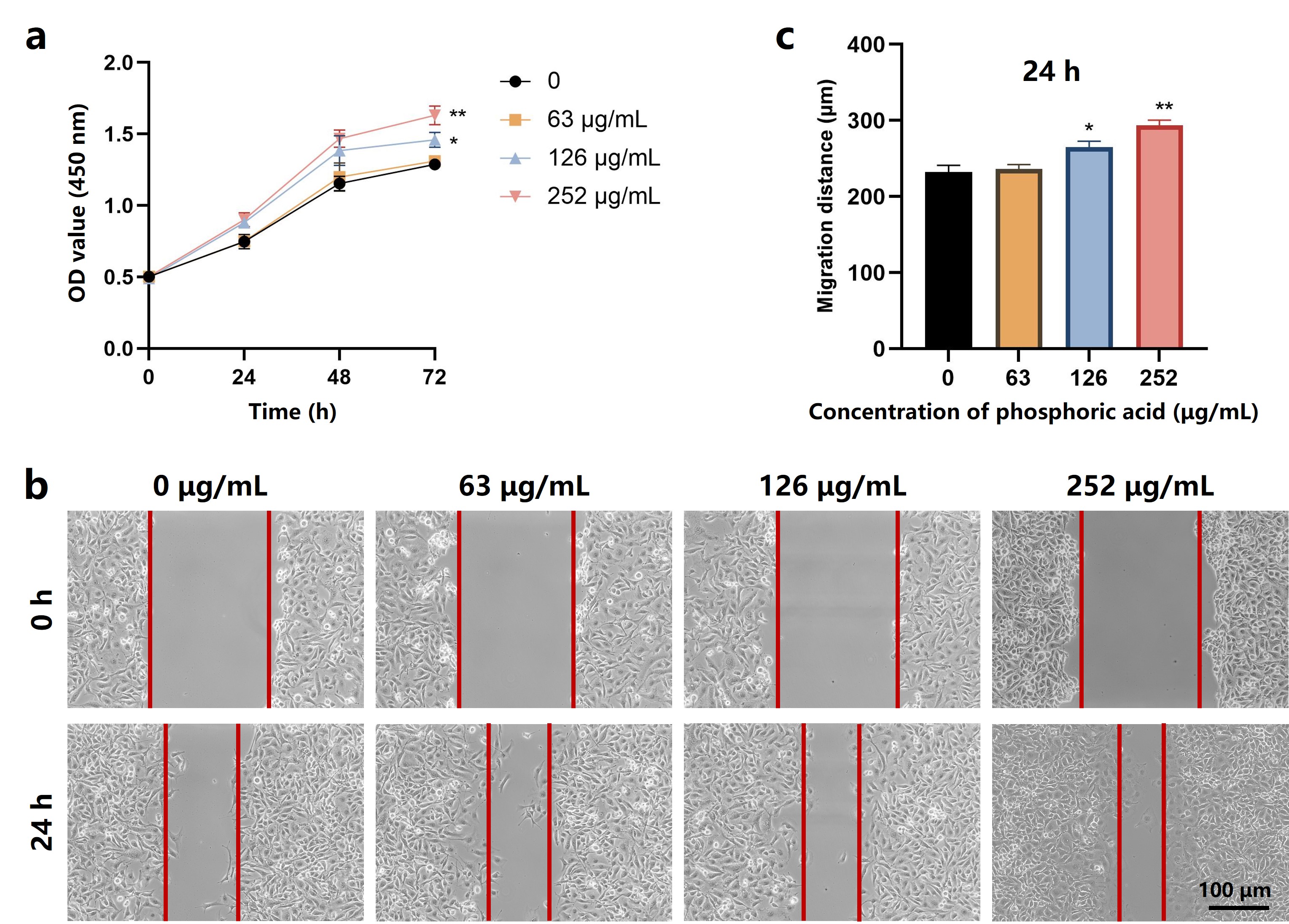


**Figure S28.** (a) Proliferation, (b) scratch experiment, (c) migration distance of HUVECs after co-incubation with different concentrations of extra phosphoric acid (the concentrations of P element were about 0, 20, 40, 80 μg mL^-1^). Data are presented as mean ± SD (*n* = 3), **p* < 0.05, ***p* < 0.01, and ****p* < 0.001.


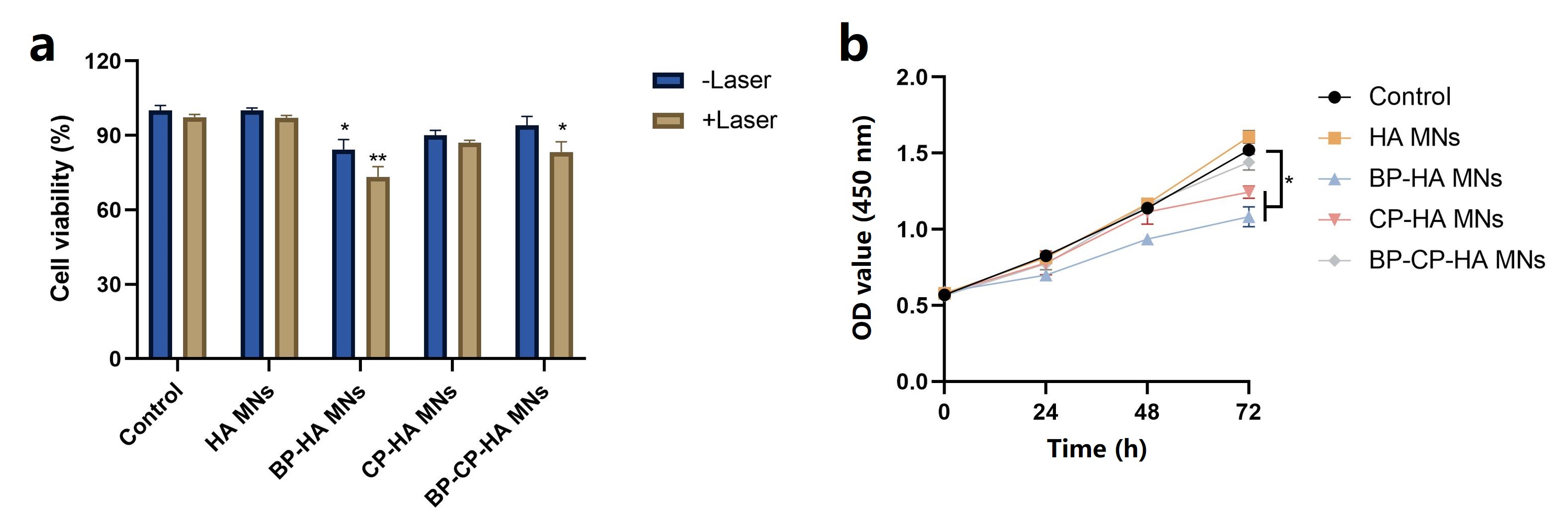


**Figure S29.** (a) Viabilities of HUVECs with or without laser irradiation. (b) OD values showing the proliferation of HUVECs after laser irradiation. Data are presented as mean ± SD (*n* = 3), **p* < 0.05, ***p* < 0.01, and ****p* < 0.001.


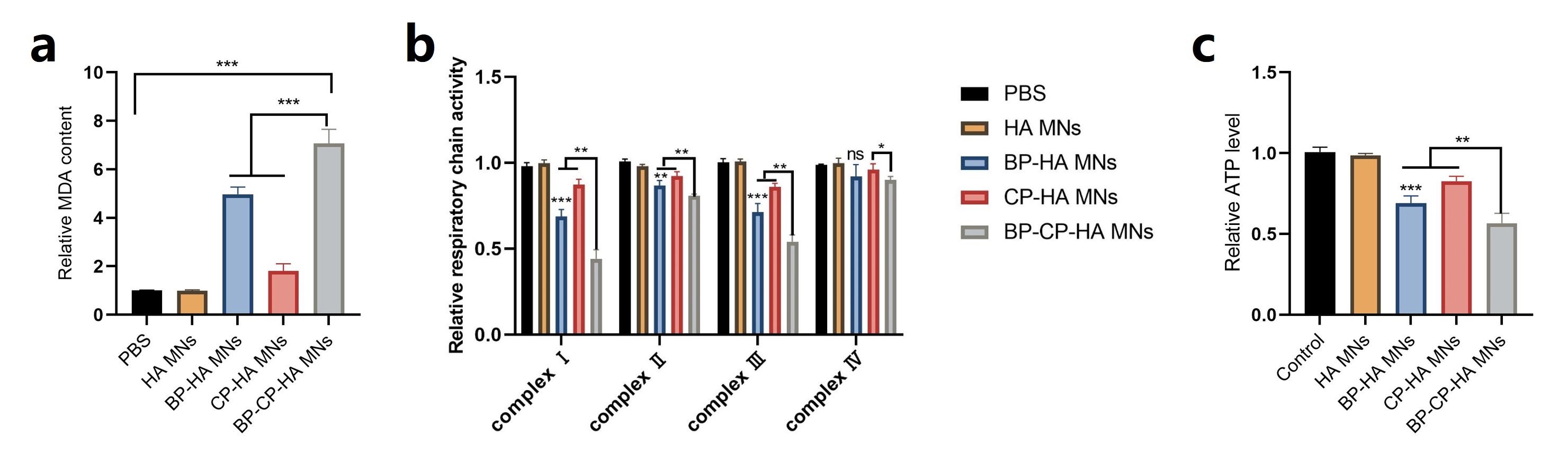


**Figure S30.** (a) MDA content, (b) respiratory chain complex activity, and (c) ATP level of MRSA in different treatment groups. Data are presented as mean ± SD (*n* = 3), **p* < 0.05, ***p* < 0.01, and ****p* < 0.001, ns: no significance.


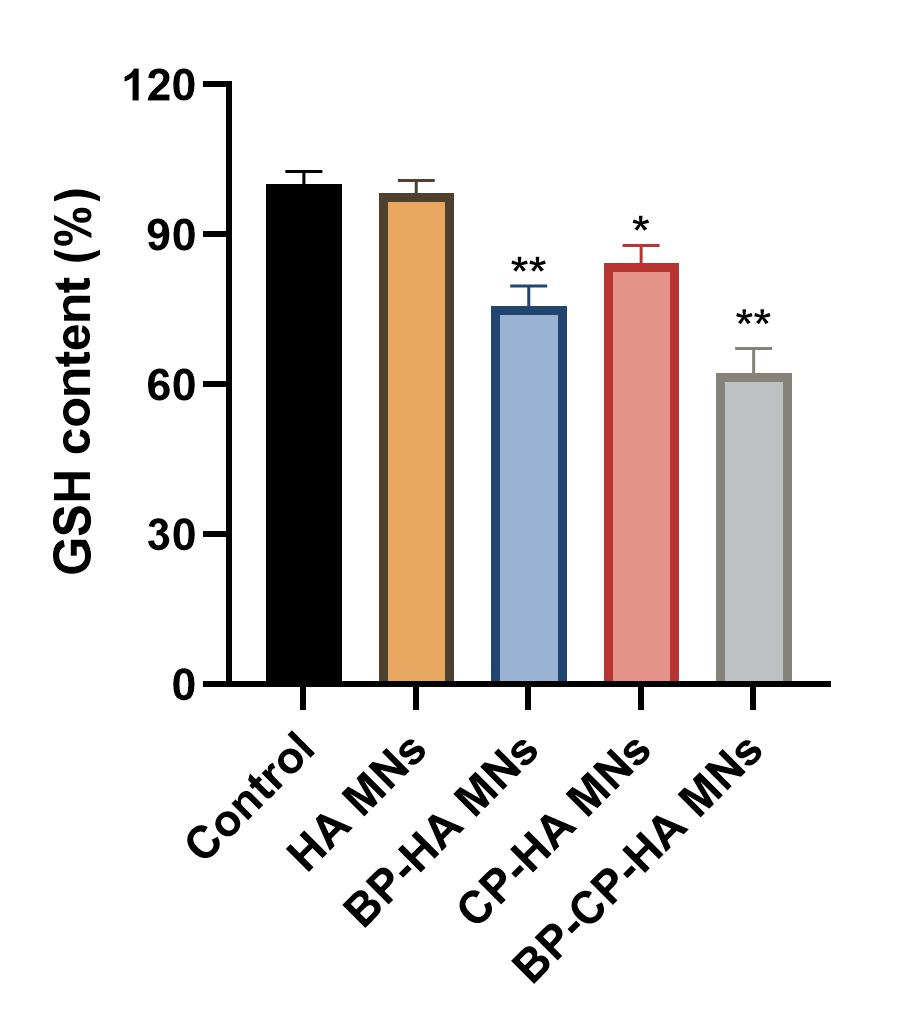


**Figure S31**. Percentage of GSH content in different groups. MRSA were incubated with normal PBS, PBS-dissolved HA MNs, BP-HA MNs, CP-HA MNs and BP-CP-HA MNs with laser irradiation (650 nm, 0.668 W cm^-2^ for 5 min). After 6 h, they were rinsed and lysed by freeze thaw method followed by the centrifugation. Then, the GSH content in the supernatant was evaluated using a GSH assay kit, and the percentage of GSH content to normal PBS group was calculated. Data are presented as mean ± SD (*n* = 3), **p* < 0.05, ***p* < 0.01, and ****p* < 0.001.


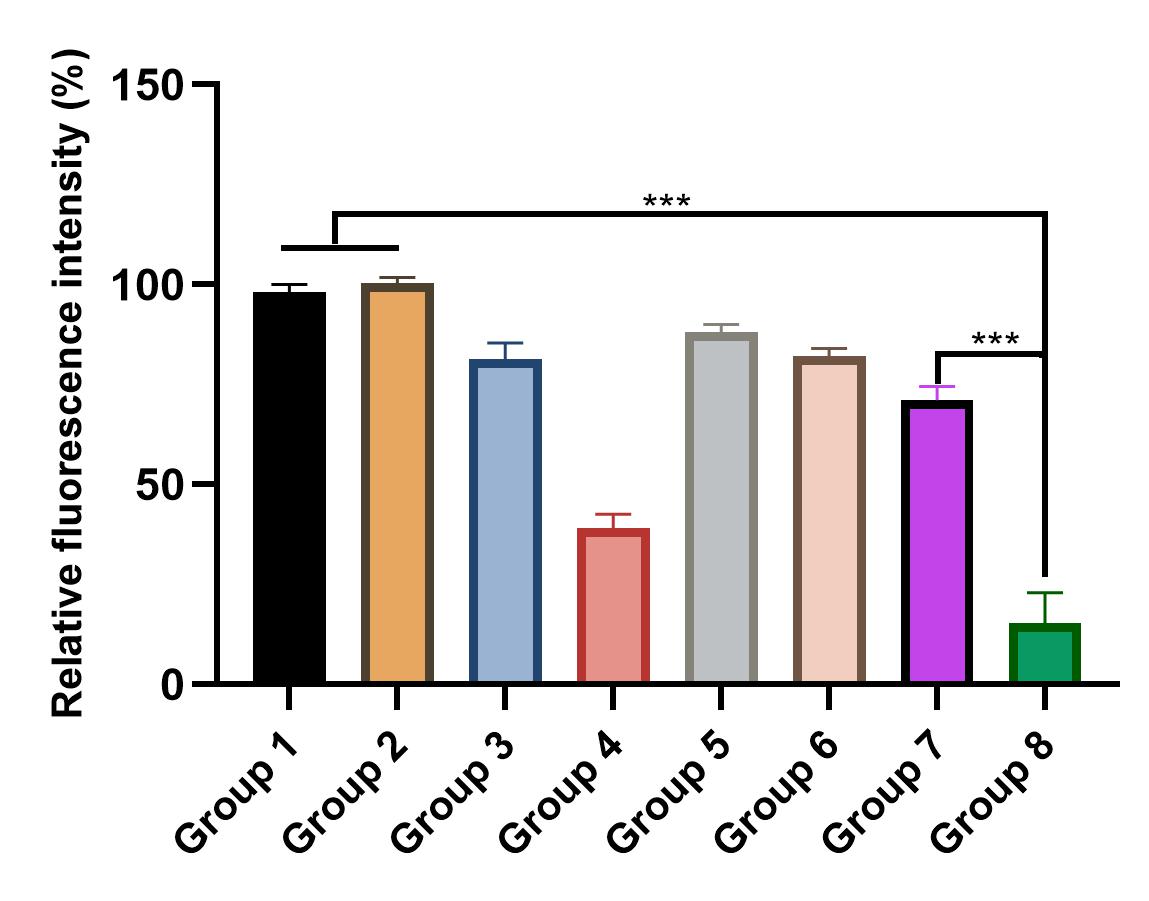


**Figure S32.** Red fluorescence intensity that represents ROS level of the wound site during the wound healing stage. Data are presented as mean ± SD (*n* = 5), **p* < 0.05, ***p* < 0.01, and ****p* < 0.001.


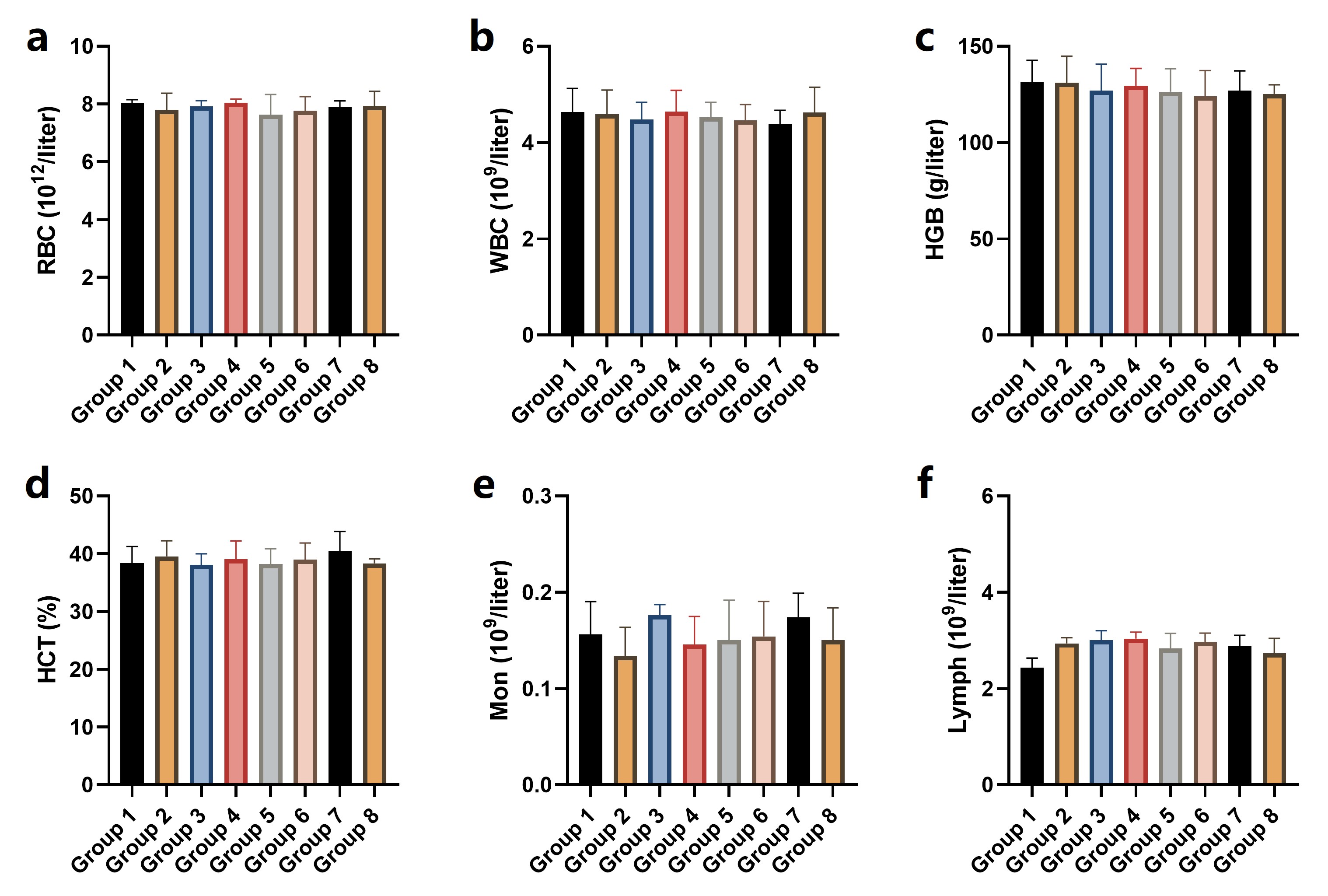


**Figure S33.** Blood routine examination, including RBC (red blood cell), WBC (white blood cell), HGB (hemoglobin), HCT (hematocrit), Mon (monocyte) and Lymph (lymphocyte) after different treatments. Data are presented as mean ± SD (*n* = 5).


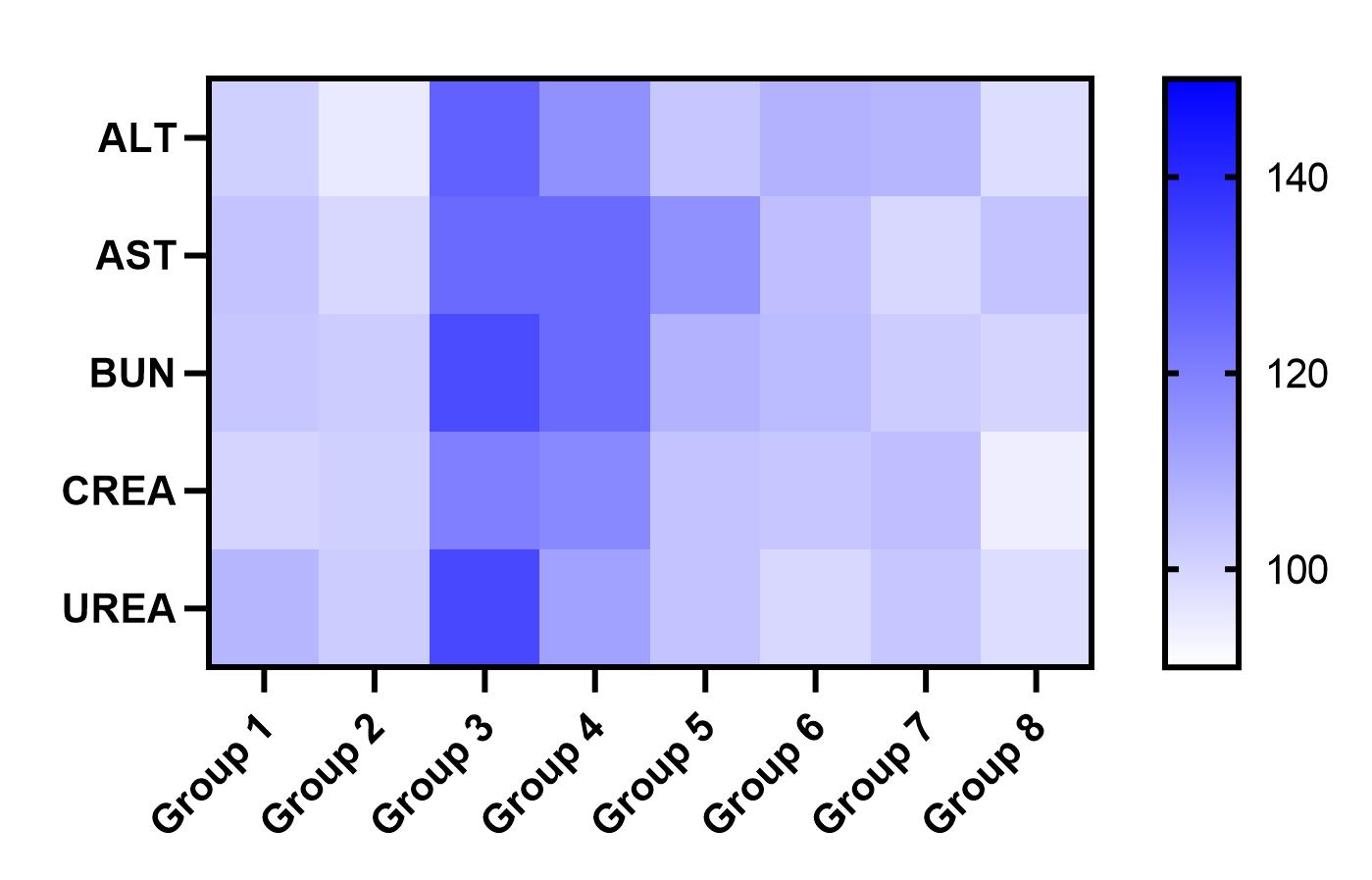


**Figure S34.** Blood biochemistry examination, including ALT (alanine aminotransferase), AST (aspartate aminotransferase), BUN (blood urea nitrogen), CREA (creatinine) and UREA after different treatments.


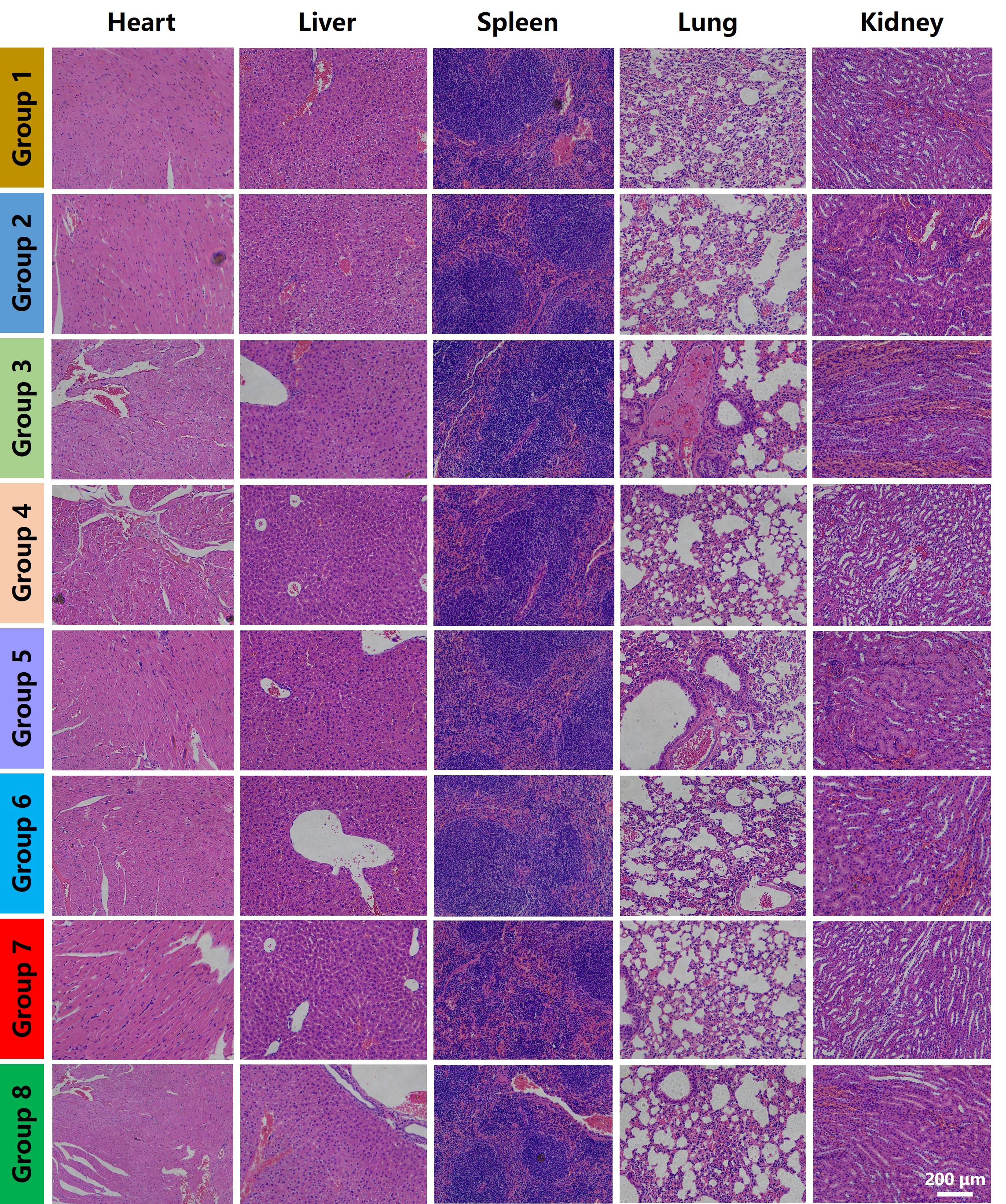


**Figure S35.** H&E staining of major organs after different treatments.


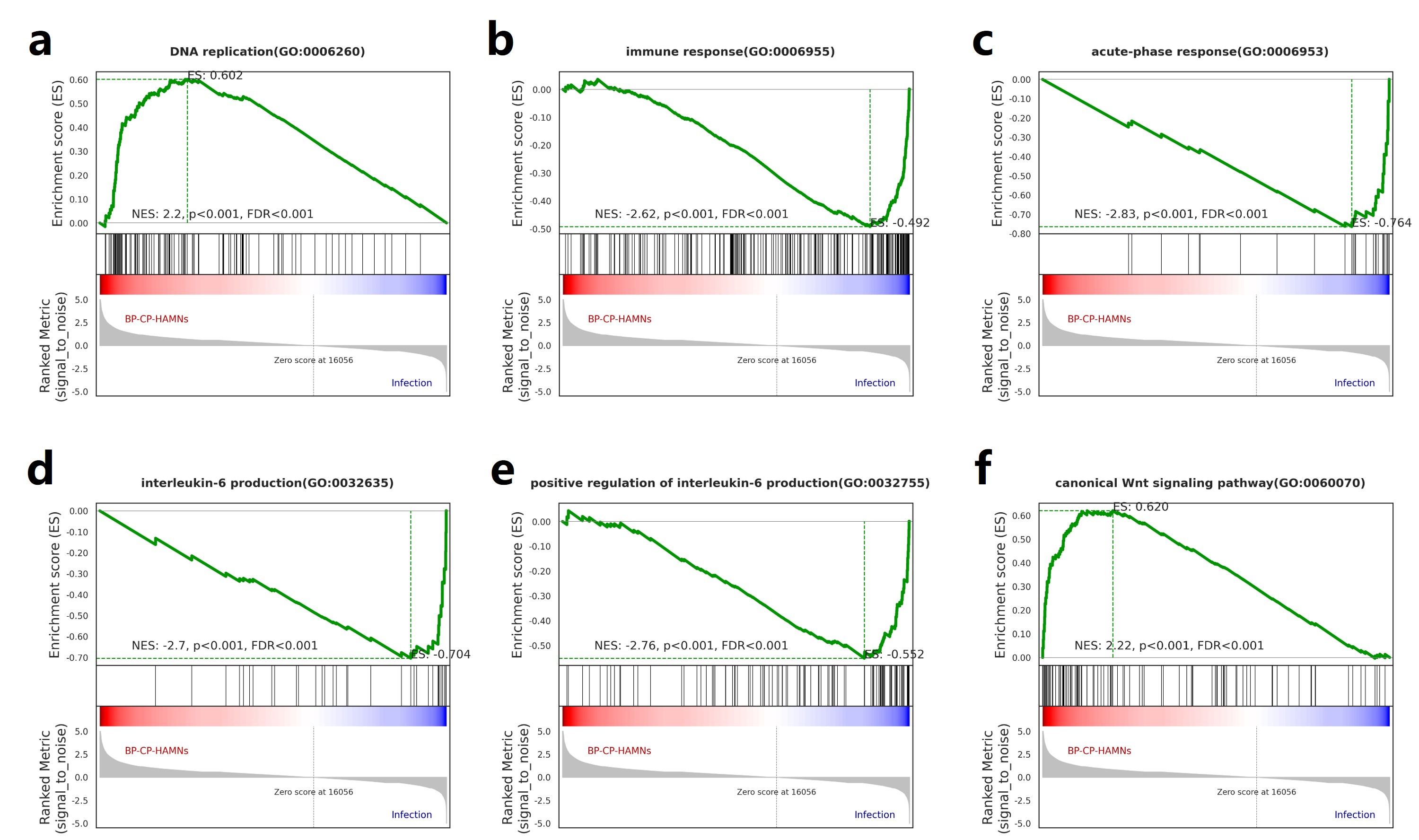


**Figure S36.** GSEA enrichment plots showing the down-regulated and up-regulated pathways after the treatment of BP-CP-HA MNs.
